# Thallium Isotopes Across the Smithian‐Spathian Boundary: Evidence for Anoxia Throughout Most of the Early Triassic

**DOI:** 10.1111/gbi.70062

**Published:** 2026-09-02

**Authors:** Sean M. Newby, Jeremy D. Owens, Seth A. Young, Øyvind Hammer

**Affiliations:** ^1^ Earth, Ocean, and Atmospheric Science Department National High Magnetic Field Laboratory, Florida State University Tallahassee Florida USA; ^2^ Department of Earth and Planetary Sciences University of Hong Kong Hong Kong Hong Kong SAR; ^3^ Natural History Museum, University of Oslo Oslo Norway

**Keywords:** Boreal Sea, end‐Permian mass extinction, Spitsbergen, trace metal geochemistry

## Abstract

Following the largest known Phanerozoic extinction, the end‐Permian mass extinction, the Early Triassic is often considered an interval of slow biotic recovery. During this time, the minor Smithian‐Spathian extinction occurred, further delaying the biotic recovery of this epoch. One mechanism linked to the slow recovery is widespread marine anoxic conditions, but the spatiotemporal changes to anoxia following the mass extinction remain poorly constrained. Here, we utilized local and global redox‐sensitive metal proxies to track variations in (de‐)oxygenation at Wallenbergfjellet, a section in Spitsbergen, Arctic Norway. Local proxies (e.g., Fe speciation and trace metal concentrations) indicate basinal anoxia and possibly euxinia prevalent in the Boreal Ocean during this interval. Importantly, thallium isotopes, a global paleoredox proxy sensitive to Mn oxide burial, have not previously been applied to the Early Triassic recovery interval that followed the end‐Permian mass extinction, though basinal restriction may limit geographic extent of interpretations. Our results show a shift in ε^205^Tl values from ~ −4.0 in the Smithian to ~ −2.0 at the Smithian‐Spathian boundary (SSB), consistent with a rapid expansion of global anoxia coincident with the boundary extinction. A rapid perturbation in thallium isotopes at the SSB (from −4.0 to −2.0 ε‐units) indicates this interval experienced an expansion of anoxic conditions that can be linked to the minor extinction event during the recovery interval following the end‐Permian mass extinction. This suggests that global anoxia persisted throughout the Early Triassic and contributed to delayed biotic recovery, with the SSB representing a major intensification of reducing conditions.

## 1 Introduction

The end‐Permian mass extinction (EPME; ~251.9 Ma) is considered the largest mass extinction of the Phanerozoic (Bond and Grasby 2017; Sepkoski 1993). The massive disruption in life marks the boundary between the Paleozoic and Mesozoic Eras and a transition from the Paleozoic fauna to the Modern fauna (McGhee et al. 2004; Sepkoski 1993). This is further compounded by a slow recovery that extended well into the Early Triassic, taking several million years, ranging from 1 to 8 Myr, to reach comparable levels of biodiversity to the pre‐EPME species richness (Brayard et al. 2006; Foster et al. 2015; Foster and Sebe 2017; Payne et al. 2004; Song et al. 2018).

Of note is the Smithian‐Spathian boundary (SSB; ~248.1 Ma, with large errors), delimiting two sub‐ages of the Olenekian Age of the Early Triassic epoch (Ogg et al. 2020; Widmann et al. 2020), and recording a minor extinction during the overall recovery phase (Galfetti, Hochuli, et al. 2007; Hammer et al. 2019; Leu et al. 2024; Song et al. 2019; Zhang et al. 2019). There is a notable loss in ammonoids that most clearly delineates this crisis interval (Galfetti, Bucher, et al. 2007; Galfetti, Hochuli, et al. 2007; Hansen et al. 2024; Zhang et al. 2019), though there is also notable faunal loss in conodonts, bivalves, and vertebrates (Hansen et al. 2018, 2024; Leu et al. 2024; Zhang et al. 2019). This crisis is also marked by latitudinal changes in species richness (Galfetti, Bucher, et al. 2007; Galfetti, Hochuli, et al. 2007; Zhang et al. 2019) and environmental shifts in the terrestrial realm as seen through changing pollen content (Blattmann et al. 2024; Galfetti, Hochuli, et al. 2007; Hansen et al. 2024; Leu et al. 2024). Though not as severe as the EPME, these changes indicate environmental degradation and faunal loss still occurred during the SSB, even as various groups were still recovering from the prior mass extinction (Foster and Sebe 2017; Galfetti, Bucher, et al. 2007).

The Smithian‐Spathian extinction is less well‐studied compared to the much larger EPME, but new investigations may provide insights into the compounding nature of these multiple crises and the mechanisms affecting biotic recovery of the Early Triassic. Most of the potential causes for both extinction events and slow recovery for this entire interval are linked to the extensive volcanism caused by the eruption of the Siberian Traps, one of the largest known flood basalts in Earth's history (Bond and Grasby 2017; Burgess et al. 2017; Song et al. 2013). The end‐Permian is directly linked to a major interval of igneous sill emplacement causing rapid climatic shifts and compounding processes (Algeo, Chen, et al. 2011; Bond and Grasby 2017; Burgess et al. 2017; Wu et al. 2023). However, the Siberian Traps volcanism is also hypothesized as a potential cause for the crisis across the SSB and the slow recovery from the prior mass extinction event (Grasby et al. 2016; Song et al. 2013). Due to this, many causal mechanisms for both extinction and lack of origination may be similar across the entire late Permian through Early Triassic, including long‐term warming (Algeo, Chen, et al. 2011; Galfetti, Bucher, et al. 2007; Song et al. 2019), short‐term cooling (Black et al. 2018; Song et al. 2019), widespread ocean anoxia (Algeo, Chen, et al. 2011; Grasby et al. 2013; Zhao et al. 2020), and volcanic metal poisoning (Grasby et al. 2016).

While igneous sill emplacement induced warming and the various proposed kill mechanisms at the EPME (Bond and Grasby 2017; Burgess et al. 2017), the SSB extinction is associated with rapid cooling following peak temperatures of the late Smithian (Song et al. 2019). This is possibly related to a specific phase of Siberian Trap volcanism more associated with atmospheric aerosols or the end of major eruptive phases for this large igneous province (Black et al. 2018; Grasby et al. 2016; Hammer et al. 2019; Song et al. 2019), either way inducing a climatic regime opposite of the EPME with volcanism‐induced cooling across the SSB rather than the warming of the Permo‐Triassic transition.

Nevertheless, widespread hypoxia and/or anoxia is often invoked as one of the primary drivers of extinction events and ecological change throughout the Phanerozoic (Bond and Grasby 2017; Feng et al. 2024; Jenkyns 2010; Sperling et al. 2022) and has been repeatedly stated as a major contributing factor to the EPME (Grasby et al. 2013; Newby et al. 2021; Song et al. 2024; Zhang, Algeo, et al. 2018; Zhang, Romaniello, et al. 2018). There is also evidence to suggest widespread anoxia was persistent through the Early Triassic and hampered the evolution and diversification of life following the EPME (Algeo, Chen, et al. 2011; Grasby et al. 2013; Song et al. 2012; Wu et al. 2024; Zhang, Romaniello, et al. 2018; Zhao et al. 2020). An expansion of anoxic conditions near the SSB may have been related to the biotic crisis (Blattmann et al. 2024; Galfetti, Hochuli, et al. 2007; Grasby et al. 2013; Hammer et al. 2019; Song et al. 2012, 2019; Wignall et al. 2016). Better constraining the extent of anoxia, especially on a global scale, and its timing to extinction events is required to better comprehend the slow recovery of the Early Triassic fauna and potential causes for loss of diversity during this interval. There are several redox proxies (e.g., δ^34^S, δ^98^Mo, δ^238^U) that are used to interpret anoxic conditions applied to this interval (Algeo, Chen, et al. 2011; Grasby et al. 2013; Song et al. 2012; Wu et al. 2024; Zhang, Romaniello, et al. 2018; Zhao et al. 2020). However, importantly, they respond to reducing conditions below the oxic‐anoxic transition, responding to changes in redox potential closer to iron and sulfate reduction zones rather than the transition out of oxic conditions where most life is impacted (Kendall et al. 2017; Lau et al. 2019; Owens 2019; Zhao et al. 2020).

### 1.1 Thallium Isotopes

Thallium isotopes are a redox proxy used to record responses in global manganese (Mn) oxide burial, and thus ocean (de−/re‐)oxygenation and have been useful when combined with additional geochemical redox proxies (Bowman et al. 2019; Kozik et al. 2022; Li et al. 2021; Newby et al. 2021; Ostrander et al. 2017, 2019; Them et al. 2018), measured as ε^205^Tl = (^205^Tl/^203^Tl_sample_ − ^205^Tl/^203^Tl_NIST‐SRM‐997_)/(^205^Tl/^203^Tl_NIST‐SRM‐997_) × 10,000 and often reported as ε‐units. More recently, a permyriad notation (‱) has been suggested (Olesen et al. 2025; Ostrander et al. 2025; Shu et al. 2025), but herein we use the historic notation. This paleoredox proxy is closely tied to the precipitation and burial of Mn oxides, specifically hexagonal birnessite (Nielsen et al. 2011, 2013; Phillips et al. 2023). Importantly, Mn oxides are a component of seafloor deposits that require available oxygen in the water column and sediments to persist through burial without undergoing dissolution (Algeo and Maynard 2004; Phillips et al. 2023; Rue et al. 1994). As one of the first elements to be affected by (de)oxygenation (Rue et al. 1994), constraining seawater Mn oxide burial at a global scale provides insights into the marine record of oxygen, documenting transitions into anoxic conditions earlier than most other paleoredox proxies. Since it is difficult to directly measure global Mn burial due to its short global residence time, the typical time Mn remains dissolved in seawater, and Mn having a single stable isotope (Owens 2019; Rehkämper et al. 2002; Rue et al. 1994; Tribovillard et al. 2006), other proxies must be utilized. Tl isotopes are ideal due to having a residence time (~18,500 years) greater than global mixing time of the ocean (~1,000–2,000 years), thus making it globally homogenous, while also having a relatively short residence time compared to other global proxies that allows it to respond to redox changes more rapidly (Nielsen et al. 2017; Owens 2019). Additionally, there is limited isotopic effect of its various sources to the ocean (rivers, mineral aerosols, sediment porewater flux, and volcanic gases) due to the overlapping similarity in their average isotopic values (all ~ −2.0 ε‐units) that minimizes any individual source from modulating seawater isotopic compositions (Nielsen et al. 2017; Owens 2019). Besides Mn oxide burial, the other primary sink, low‐temperature alteration of oceanic crust, has minimal fractionation from seawater (−1.2 ε‐units) while also only occurring on multi‐million year timescales that are beyond the scope of most short‐term events (Prytulak et al. 2013; Shu et al. 2022).

Therefore, Mn oxide formation and burial, which induce a positive fractionation (+16.0 ε‐units) on Tl isotopes from global seawater values (Nielsen et al. 2006, 2013), is the primary control on the seawater isotopic value (modern‐day oxic seawater value is −6.0 ε‐units). Without Mn oxide formation, seawater Tl isotopic values would be similar to source values (−2.0 ε‐units); therefore, allowing for the tracking of global changes in oxic seafloor through time via seawater Tl isotopes (Chen et al. 2021; Owens et al. 2017). Fluctuations in these values have been used to interpret redox changes across several anoxic intervals in geologic history (Bowman et al. 2019; Kozik et al. 2022; Li et al. 2021; Newby et al. 2021; Ostrander et al. 2017, 2019; Them et al. 2018). For the most accurate reconstruction of ancient seawater isotopic compositions, sediments buried under anoxic to euxinic (anoxic with sulfidic water column) conditions should be utilized as they capture overlying seawater values due to the minimal fractionation during adsorption to pyrite formed under these redox conditions (Fan, Nielsen, et al. 2020; Owens et al. 2017; Wang et al. 2022). Euxinic basins such as the restricted Cariaco Basin have sufficient open ocean connection to record these seawater values (~ −6.0), though caution should be taken as the significantly more restricted Black Sea also is euxinic but only records source values (~ −2.0) due to lack of sufficient ocean mixing (Owens et al. 2017). Thus, the use of additional redox proxies that track local geochemistry such as iron (Fe) speciation (Raiswell et al. 2018) and trace metal concentrations (Algeo and Maynard 2004), particularly Mn to ensure limited local fractionation from Mn oxides, are needed to confirm the efficacy of Tl isotopes at recording seawater compositions. Following determination of local anoxia to euxinia, these sediments can range from −6.0 for oxic seawater to −2.0 for widespread marine anoxia (source‐dominated), providing a record of dynamic shifts in marine redox conditions between oxic and anoxic conditions on a global scale. Studying the Wallenbergfjellet section of Svalbard can, thus, be useful due to previous work indicating locally anoxic conditions across much of the Smithian and Spathian marine strata deposited at this site (Blattmann et al. 2024; Hammer et al. 2019) and generally anoxic conditions throughout the Boreal Sea (Grasby et al. 2016; Wignall et al. 2016), allowing for more accurate Tl isotopic reconstruction.

### 1.2 Earliest Triassic Thallium Isotopes

Previously published Tl isotope data on the EPME (Newby et al. 2021) provide a framework for addressing the long‐term. Early Triassic paleoenvironmental reconstruction using this proxy. Importantly to this study, following the EPME, the Tl isotopes remain at values that indicate highly anoxic conditions (−2.0) throughout the remainder of the sections well into the Griesbachian subage of the Early Triassic (Newby et al. 2021), matching other global redox proxies applied to the Early Triassic (Zhang, Romaniello, et al. 2018; Zhao et al. 2020). As Tl isotopes react to changes in marine oxygen sooner than many other global redox proxies, utilizing this proxy across the Early Triassic in conjunction with the work done with other global redox proxies (Song et al. 2019; Zhang, Romaniello, et al. 2018; Zhao et al. 2020) can better constrain the mechanisms that induced both the slow recovery of this interval and the minor extinction at the SSB.

## 2 Materials and Methods

### 2.1 Geologic Setting

**FIGURE 1:**
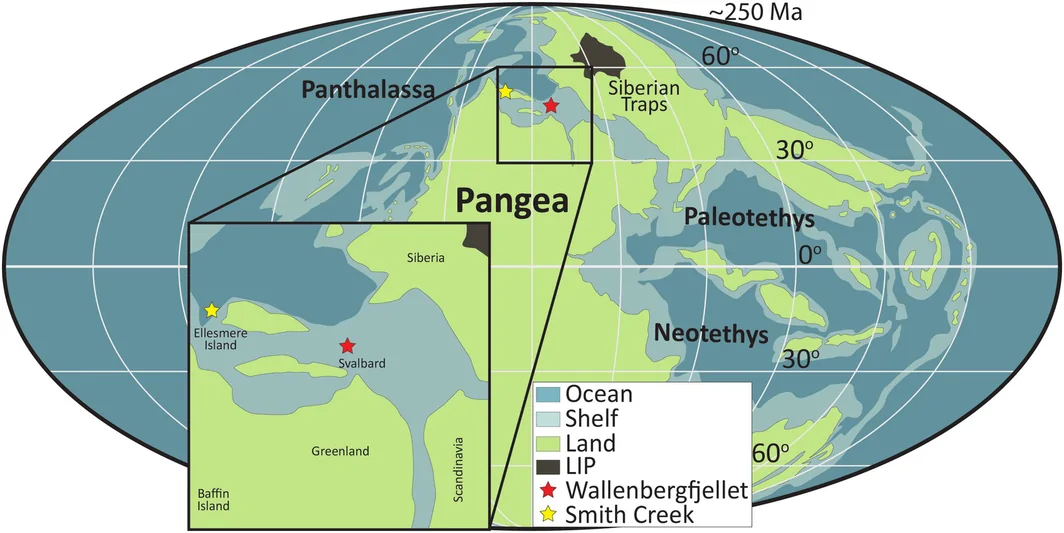
A paleogeographic map of the Early Triassic (~250 Ma) with sampling location at Wallenbergfjellet (Hammer et al. 2019; Hansen et al. 2018) and the well‐studied type section, Smith Creek (Grasby et al. 2013, 2016), also found within the Boreal Ocean. Map modified from Blakey (2016) and Torsvik and Cocks (2016). LIP = large igneous province.

The Wallenbergfjellet section in Spitsbergen, Svalbard is dominated by organic‐rich mudstones of the Lower Triassic, primarily being laminated shales and some grey siltstones, with minor carbonates (Hammer et al. 2019; Hansen et al. 2018, 2024). This section is within the Boreal Ocean, which also contains the well‐studied Sverdrup Basin where the type section at Smith Creek for the Smithian (Figure 1) and the Spath Creek type section for the Spathian are found (Grasby et al. 2013). These sections are all found at middle northern paleolatitudes (Figure 1). The Vikinghøgda Formation is a biostratigraphically well‐constrained, using conodonts and ammonoids, and lithologically well‐correlated formation, supplemented with carbon chemostratigraphy, that was deposited in an offshore setting during the Early Triassic and can be found in central and eastern Svalbard, including at Wallenbergfjellet (Blattmann et al. 2024; Hansen et al. 2018, 2024; Leu et al. 2024), here representing a gently sloping marine ramp (Wignall et al. 2016). The Vikinghøgda Formation can be further divided at Wallenbergfjellet into the lower Lusitaniadalen Member and upper Vendomdalen Member, approximately representing the Smithian and Spathian, respectively (Hansen et al. 2018). The lowermost Deltadalen Member (Induan) is not exposed at this locality. All sample heights are presented in relation to this contact between the Lusitaniadalen and Vendomdalen Members and the boundary between substages. Previous work has generated detailed ammonoid and conodont biostratigraphy to confirm the ages for this section and the nearby and equivalent Stensiöfjellet section, albeit limited fossil abundance through much of the Vendomdalen Member required use of carbon chemostratigraphy to aid in age constraints (Hammer et al. 2019; Hansen et al. 2018; Leu et al. 2024). The Lusitaniadalen Member included *Arctoceras* cf. *blomstrandi* and *Juvenites spathi* of the *Euflemingites romunderi* ammonoid biozone lower in the section and the *Wasatchites tardus* ammonoid biozone immediately below the member boundary, with *Scythogondolella milleri* and *Scythogondolella mosheri* conodont biozones of the late Smithian (Hammer et al. 2019; Hansen et al. 2018; Leu et al. 2024). Above this in the Vendomdalen Member were the *Bajarunia euomphalab* ammonoid biozone immediately above the boundary and the *Keyserlingites subrobustus* ammonoid biozone representing the remainder of this formation, matched with the *Novispathodus pingdingshanensis* conodont biozone of the early Spathian (Hammer et al. 2019; Hansen et al. 2018; Leu et al. 2024). Additionally, there is a possible gap in the ammonoid biostratigraphic record at the boundary between these members that strongly suggests loss of some of the latest Smithian (Hammer et al. 2019; Hansen et al. 2024), potentially coeval with similar sedimentary gaps in the Sverdrup Basin and Boreal Ocean as a whole (Grasby et al. 2013; Leu et al. 2024).

Shale samples dug from below the weathered outcrop surface were previously collected from the Wallenbergfjellet section (Hammer et al. 2019), obtaining material starting from slightly below the boundary between the two noted members of the Vikinghøgda Formation upwards to the base of the overlying formation, the Botneheia Formation that represents the Anisian and Ladinian stages. These samples were collected at 2 m intervals except within the vicinity of the boundary between members, where collection was at 1 m intervals (Hammer et al. 2019). Between 5 and 10 g of sample were powdered using a tungsten carbide ball mill for all the geochemical analyses.

### 2.2 Geochemical Analyses

A split of each powdered sample (~100 mg) was used for sequential Fe speciation, an independent method for determining local redox conditions (Raiswell et al. 2018). This involves sequential extraction of reactive Fe phases in carbonate (Fe_carb_), oxides (Fe_ox_), and magnetite (Fe_mag_), a separate extraction for reactive Fe in pyrite (Fe_py_), and a total digestion method to get total Fe (Fe_T_). The sequential extraction procedure follows previous studies (Poulton and Canfield 2005) and utilizes pH buffered solutions of sodium acetate for Fe_carb_ extraction, dithionite for Fe_ox_ extraction, and ammonium oxalate for Fe_mag_ extraction. Concentrations of all of these aliquots were then measured on an Agilent 7500cs quadrupole inductively coupled plasma mass spectrometer (ICP‐MS) with duplicate samples run with less than 5% deviation from each other (Table S1).

The last portion of Fe speciation required the isolation of Fe_py_ using another sample split (~1 g). This material underwent chromium‐reducible sulfide extraction modified from previous studies (Brüchert and Pratt 1996; Lefticariu et al. 2006). A chromium chloride solution was reacted with the samples in a pure nitrogen atmosphere to produce hydrogen sulfide gas, which reacted with silver nitrate to form silver sulfide. This residue was then weighed and stoichiometrically used to determine the original concentration of Fe_py_. This could be used in conjunction with the other portions of Fe speciation to determine local redox conditions (Raiswell et al. 2018). Highly reactive phases (Fe_HR_) are composed of Fe_carb_ + Fe_ox_ + Fe_mag_ + Fe_py_. A Fe_HR_/Fe_T_ ratio of greater than 0.38 is used to determine locally anoxic conditions, while values greater than 0.22 are typically inferred as being possibly anoxic, and less than 0.22 suggesting oxic deposition. Samples with Fe_HR_/Fe_T_ above 0.38 can then be interpreted for the type of anoxic deposition using Fe_py_/Fe_HR_. A Fe_py_/Fe_HR_ ratio of greater than 0.8 determines locally euxinic conditions, while values greater than 0.6 indicate possibly euxinic, though a threshold of 0.7 has previously been used as well, and any ratio less than 0.6 (0.7) is assumed to be ferruginous (Benkovitz et al. 2020). Additionally, the Fe_T_/Al ratio, which can track potential Fe enrichments in sediments compared to typical oxic marine mud composition around 0.55, was calculated (Raiswell et al. 2018).

Elemental concentrations were determined using another powdered split (~100 mg). These were ashed in crucibles at high temperatures (~400°C) in an oven for ~12 h. Then the ashed samples were transferred to clean Teflon beakers to react with multiple mixtures containing hydrofluoric, hydrochloric, and/or nitric acids until the samples were completely dissolved. These solutions were then measured on the same Agilent 7500cs quadrupole ICP‐MS. This provided bulk concentrations for aluminum (Al), vanadium (V), Fe_T_, Mn, Mo, and U. Enrichment factors (EF) were calculated as ME_EF_ = (ME/Al)_sample_/(ME/Al)_PAAS_, where ME represents a specific metal element and PAAS is post‐Archean average Australian shale (Mclennan 2001; Taylor and Mclennan 1985). The USGS georeferenced standards SDO‐1, SCo‐2, and SGR‐1 were also measured, having an average of less than 10% deviation from the reported concentration range for measured elements of Al, V, Mn, Fe, Mo, and U (Table S2).

Previously described and now standard methods were used to extract Tl for isotopic analysis (Nielsen et al. 2009; Ostrander et al. 2017). For a simplified explanation, this involved a hot overnight leach of samples in nitric acid, dissolution of remaining organic matter in a mixed nitric‐hydrochloric acid solution, and column chemistry. Brominated water was utilized to oxidize the Tl before column chemistry, with various nitric and hydrochloric acid washes before elution using a reducing sulfur dioxide solution. Samples were then analyzed on the same Agilent 7500cs quadrupole ICP‐MS for concentrations. Samples were then spiked with NIST 981 Pb before being analyzed on a Neptune multi‐collector ICP‐MS using an Aridus II autosampler for isotopic compositions. These were ratioed to the NIST‐SRM‐997 metal standard and compared to Geostandard SCo‐1, which has a long‐term precision of ε^205^Tl = −3.0 ± 0.3 (2σ) (Nielsen et al. 2017; Ostrander et al. 2017; Owens 2019; Owens et al. 2017), with our analyses determining −2.8 ± 0.2 (2*n*) for SCo‐1. The two‐standard deviations for all samples were increased to a minimum of ±0.3 to match this long‐term precision.

## 3 Results

**FIGURE 2:**
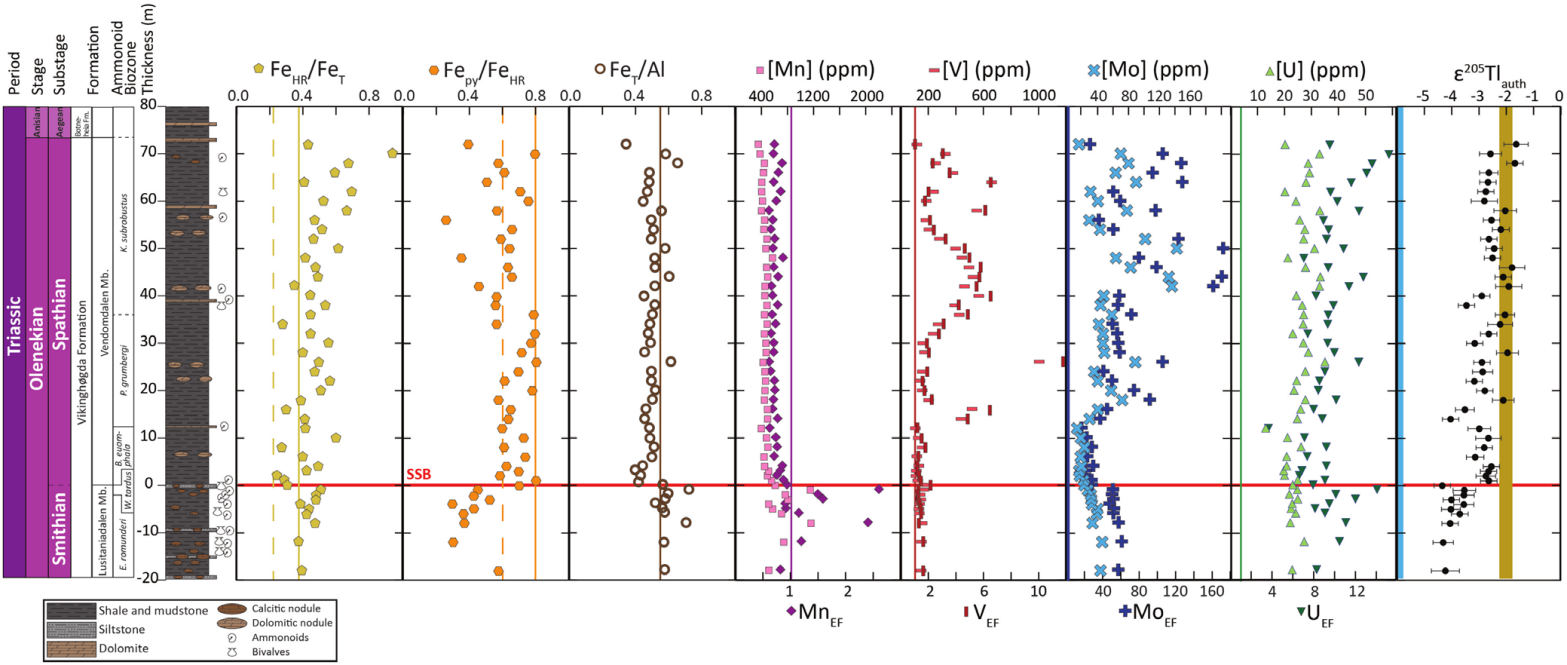
Lithology, biostratigraphy and geochemistry of Wallenbergfjellet. Lithology and biostratigraphy based on previous studies of the Vikinghøgda Formation and Wallenbergfjellet section (Hammer et al. 2019; Hansen et al. 2018). Key for stratigraphic column and fossils included. Geochemistry includes Fe species, trace metal concentrations, and thallium isotopes. The symbol and color associated with each element/ratio shown are included adjacent to the given element/ratio. For trace metals, concentrations are listed on top and enrichment factors listed on the bottom. Vertical gold dashed line (0.22) in Fe_HR_/Fe_T_ species plot denotes the lower bound for possibly anoxia, with vertical gold solid line (0.38) denoting lower bound for definite anoxia (Poulton and Canfield 2005; Raiswell et al. 2018). The vertical orange dashed line (0.6) in Fe_py_/Fe_HR_ species plot denotes the lower bound for possibly euxinia, with a vertical orange solid line (0.8) denoting the lower bound for definite euxinia. The vertical brown line in Fe_T_/Al represents average oxic shale composition (0.55). Vertical colored lines in trace metal concentration plots represent PAAS values for the respectively colored element related to the top axis, specifically purple for Mn (852 ppm), maroon for V (107 ppm), dark blue for Mo (1.5 ppm), and green for U (2.8 ppm) or enrichment factor of 1 compared to the enrichment factor axis on the bottom (Mclennan 2001; Taylor and Mclennan 1985). The vertical light blue bar in ε^205^Tl represents modern oxic seawater values (−6.0), with the average modern source values (−2.0) indicated with a vertical brown bar (Owens 2019). Horizontal red line marks the Smithian‐Spathian boundary.

Geochemical data for Fe species concentrations and redox‐sensitive trace metal concentrations (Mn, V, Mo, and U) were measured (Table 1) as well as various geochemical ratios and Tl isotopes (Table 2), organized in stratigraphic order (Figure 2). The Fe_HR_/Fe_T_ ratios are all above the 0.22 threshold for possible anoxia, with most (83%) above the 0.38 threshold for anoxia. There is a general increasing trend going up the section, with larger variability as well. The Fe_py_/Fe_HR_ ratios are between 0.26 and 0.94, but only half (54%) are greater than the possibly sulfidic threshold at 0.6, and only four samples are at or above the 0.8 ratio for sulfidic. There is a rapid increase in Fe_py_/Fe_HR_ ratio just prior to the SSB, which maintains values above 0.5 throughout most of the remaining section. Also measured were Fe_T_/Al ratios, which are primarily around modern marine mud values (Raiswell et al. 2018), with only 8 samples of 48 outside of one standard deviation (±0.11) from typical values and a full range of only 0.40 to 0.72.

**TABLE 1:** Geochemical concentrations collected for the Wallenbergfjellet Section, Svalbard.

Sample ID	Elevation (m)	Iron species concentrations					Trace metal concentrations						
		Fe_carb_ (Wt.%)	Fe_oxide_ (Wt.%)	Fe_mag_ (Wt.%)	Fe_py_ (Wt.%)	Fe_HR_ (Wt.%)	Al (Wt. %)	V (ppm)	Mn (ppm)	FeT (Wt. %)	Mo (ppm)	U (ppm)	Tl_auth_ (ppb)
WAL B‐18m	−18	0.09	0.57	0.07	1.00	1.74	7.66	160.00	517.77	4.42	44.46	22.63	386.55
WAL B‐12m	−12	0.10	0.92	0.10	0.49	1.61	7.57	158.12	747.65	4.30	46.74	26.98	293.48
WAL B‐8m	−8	0.26	0.76	0.23	0.73	1.98	5.87	128.24	1169.37	4.18	34.29	21.93	210.47
WAL B‐6m	−6	0.10	0.77	0.29	0.66	1.81	7.52	145.70	714.00	4.31	39.72	23.90	277.36
WAL B‐5m	−5	0.06	0.79	0.23	0.80	1.88	7.63	142.88	575.68	4.31	40.72	22.23	146.17
WAL B‐4m	−4	0.05	0.68	0.25	0.41	1.39	6.88	130.96	515.87	3.59	33.49	22.66	326.77
WAL B‐3m	−3	0.14	0.53	0.16	0.90	1.72	6.18	123.83	811.22	3.62	32.61	24.64	202.69
WAL B‐2m	−2	0.15	0.73	0.14	0.76	1.77	6.24	123.72	771.68	3.69	32.06	21.61	212.98
WAL B‐1m	−1	0.26	0.65	0.18	0.88	1.97	5.37	127.01	1158.62	3.86	28.11	24.52	165.16
WAL B 0m	0	0.05	0.29	0.07	0.97	1.38	7.99	215.49	616.30	4.51	23.61	22.84	532.96
WAL B 1m	1	0.04	0.10	0.05	0.75	0.94	7.76	144.41	560.35	3.26	22.69	24.65	471.62
WAL B 2m	2	0.03	0.26	0.05	0.48	0.82	7.96	131.60	496.70	3.37	17.76	19.86	399.22
WAL B 3m	3	0.04	0.28	0.07	0.89	1.28	7.62	119.67	513.55	3.04	17.02	19.54	281.34
WAL B 4m	4	0.03	0.37	0.12	0.86	1.38	6.42	121.02	452.48	2.80	19.97	20.64	513.55
WAL B 6m	6	0.03	0.29	0.08	1.11	1.51	7.62	127.38	443.48	3.80	18.76	20.63	374.12
WAL B 8m	8	0.05	0.29	0.10	0.68	1.12	8.05	175.40	503.41	4.13	23.76	25.86	811.50
WAL B 10m	10	0.03	0.48	0.12	1.70	2.33	7.95	147.55	482.08	3.87	19.05	20.92	465.78
WAL B 12m	12	0.02	0.49	0.11	0.94	1.57	7.77	119.81	400.79	3.74	14.27	13.01	713.57
WAL B 14m	14	0.04	0.43	0.07	0.94	1.48	7.89	480.79	498.79	3.60	30.51	24.52	2035.60
WAL B 16m	16	0.04	0.30	0.10	0.80	1.24	8.95	643.00	497.50	4.14	41.16	25.72	2554.11
WAL B 18m	18	0.04	0.52	0.08	0.88	1.53	7.89	222.68	449.14	3.94	72.58	27.42	1387.88
WAL B 20m	20	0.04	0.33	0.09	1.61	2.07	7.77	171.46	458.41	4.04	58.23	23.31	674.47
WAL B 22m	22	0.04	0.63	0.19	1.38	2.25	8.01	158.65	467.45	3.98	41.10	24.26	546.47
WAL B 24m	24	0.04	0.39	0.17	1.40	2.01	8.62	192.74	456.99	4.26	36.28	27.39	576.93
WAL B 26m	26	0.05	0.31	0.14	2.09	2.59	8.47	1176.96	429.66	5.20	89.51	34.51	7234.41
WAL B 28m	28	0.07	0.27	0.08	1.07	1.50	8.31	203.70	476.51	3.76	49.53	28.49	666.98
WAL B 30m	30	0.06	0.31	0.13	1.70	2.21	8.22	189.34	471.97	3.97	47.69	26.69	660.32
WAL B 32m	32	0.08	0.21	0.07	1.42	1.79	8.39	276.60	449.61	4.02	47.77	22.84	1398.97
WAL B 34m	34	0.07	0.30	0.11	0.62	1.11	8.18	310.82	492.27	3.99	42.43	26.60	1203.24
WAL B 36m	36	0.08	0.23	0.08	1.46	1.85	8.21	485.14	451.35	4.15	59.23	26.79	2198.21
WAL B 38m	38	0.04	0.63	0.28	1.20	2.16	7.73	420.86	489.04	4.03	44.56	26.28	2452.00
WAL B 40m	40	0.04	0.56	0.12	0.93	1.65	8.21	646.26	456.26	3.70	48.55	24.14	2120.59
WAL B 42 m	42	0.02	0.53	0.28	0.70	1.53	8.54	549.00	459.05	4.41	136.85	32.58	2242.84
WAL B 44m	44	0.07	0.48	0.23	1.51	2.29	7.83	570.43	500.75	4.69	132.69	32.85	2483.22
WAL B 46m	46	0.03	0.47	0.28	1.32	2.09	8.42	579.26	483.03	4.36	83.51	27.48	726.22
WAL B 48 m	48	0.13	0.66	0.33	0.61	1.72	8.04	495.42	575.00	4.13	64.50	21.03	4061.34
WAL B 50m	50	0.04	0.79	0.24	1.93	3.00	8.37	464.22	472.03	4.85	143.13	30.72	2217.47
WAL B 52m	52	0.04	0.61	0.13	1.13	1.91	8.33	325.54	489.12	4.12	102.30	26.80	1255.32
WAL B 54m	54	0.03	0.52	0.19	1.43	2.17	8.31	239.62	442.43	4.21	43.87	27.21	3303.65
WAL B 56m	56	0.03	1.20	0.16	0.49	1.88	8.08	211.34	453.61	3.98	29.95	25.36	1565.64
WAL B 58m	58	0.04	1.01	0.24	1.69	2.99	7.99	612.50	404.38	4.46	78.59	32.64	9801.25
WAL B 60m	60	0.06	0.26	0.08	1.22	1.62	6.85	179.11	419.63	3.08	41.20	24.01	2278.71
WAL B 62m	62	0.09	0.43	0.04	1.39	1.95	6.01	200.65	408.06	2.82	31.65	19.99	1112.07
WAL B 64m	64	0.03	0.53	0.14	0.71	1.40	7.13	652.04	408.62	3.45	90.56	27.65	4967.20
WAL B 66m	66	0.04	0.58	0.13	1.18	1.93	6.75	357.02	434.44	3.25	64.09	28.92	3189.18
WAL B 68m	68	0.06	0.93	0.22	1.63	2.84	6.42	231.57	448.73	4.20	80.81	28.44	2250.20
WAL B 70m	70	0.05	0.56	0.14	2.92	3.66	6.67	301.58	381.28	3.89	70.66	32.55	3205.33
WAL B 72m	72	0.03	0.46	0.06	0.36	0.91	6.07	103.91	354.45	2.09	16.75	20.05	208.12

**TABLE 2:** Geochemical ratios collected for Wallenbergfjellet Section, Svalbard, including thallium isotopes.

Sample ID	Elevation (m)	Iron species ratios			Trace metal enrichment factors				(Mo_EF_/U_EF_)_sample_/(Mo_EF_/U_EF_)_sea_ ^a^	Thallium isotopes	
		Fe_HR_/Fe_T_	Fe_py_/Fe_HR_	Fe_T_/Al	Mn_EF_	V_EF_	U_EF_	Mo_EF_		ε^205^Tl_auth_	Error (2sd)
WAL B‐18m	−18	0.39	0.58	0.58	0.79	1.39	9.53	58.07	1.85	−4.22	0.51
WAL B‐12m	−12	0.37	0.30	0.57	1.16	1.39	11.49	61.73	1.63	−4.30	0.37
WAL B‐8m	−8	0.47	0.37	0.71	2.34	1.46	12.04	58.37	1.47	−4.05	0.30
WAL B‐6m	−6	0.42	0.36	0.57	1.11	1.29	10.25	52.81	1.56	−3.69	0.31
WAL B‐5m	−5	0.44	0.43	0.56	0.89	1.25	9.40	53.36	1.72	−4.02	0.35
WAL B‐4m	−4	0.39	0.30	0.52	0.88	1.27	10.63	48.68	1.39	−3.55	0.37
WAL B‐3m	−3	0.48	0.52	0.59	1.54	1.34	12.86	52.77	1.24	−4.00	0.30
WAL B‐2m	−2	0.48	0.43	0.59	1.45	1.32	11.17	51.38	1.39	−3.54	0.38
WAL B‐1m	−1	0.51	0.45	0.72	2.53	1.58	14.72	52.30	1.08	−3.53	0.41
WAL B 0m	0	0.31	0.70	0.57	0.91	1.80	9.22	29.56	0.97	−4.34	0.30
WAL B 1m	1	0.29	0.80	0.42	0.85	1.24	10.25	29.24	0.86	−2.63	0.30
WAL B 2m	2	0.24	0.58	0.42	0.73	1.10	8.05	22.31	0.84	−2.74	0.36
WAL B 3m	3	0.42	0.70	0.40	0.79	1.05	8.27	22.34	0.82	−2.64	0.30
WAL B 4m	4	0.49	0.62	0.44	0.83	1.26	10.37	31.10	0.91	−2.54	0.30
WAL B 6m	6	0.40	0.73	0.50	0.68	1.11	8.73	24.60	0.85	−3.13	0.32
WAL B 8m	8	0.27	0.61	0.51	0.73	1.45	10.37	29.53	0.86	−2.80	0.30
WAL B 10m	10	0.60	0.73	0.49	0.71	1.24	8.49	23.95	0.86	−2.65	0.47
WAL B 12m	12	0.42	0.60	0.48	0.61	1.03	5.40	18.38	1.03	−2.98	0.42
WAL B 14m	14	0.41	0.64	0.46	0.74	4.06	10.02	38.64	1.17	−4.03	0.30
WAL B 16m	16	0.30	0.65	0.46	0.65	4.79	9.27	46.01	1.50	−3.51	0.35
WAL B 18m	18	0.39	0.58	0.50	0.67	1.88	11.21	92.00	2.49	−2.10	0.40
WAL B 20m	20	0.51	0.78	0.52	0.69	1.47	9.68	74.94	2.35	−2.78	0.30
WAL B 22m	22	0.57	0.61	0.50	0.69	1.32	9.77	51.34	1.59	−3.16	0.30
WAL B 24m	24	0.47	0.70	0.49	0.62	1.49	10.24	42.08	1.24	−2.86	0.37
WAL B 26m	26	0.50	0.81	0.61	0.60	9.27	13.15	105.73	2.44	−2.88	0.30
WAL B 28m	28	0.40	0.72	0.45	0.67	1.63	11.06	59.58	1.63	−1.95	0.40
WAL B 30m	30	0.56	0.77	0.48	0.67	1.54	10.48	58.04	1.68	−3.15	0.30
WAL B 32m	32	0.44	0.80	0.48	0.63	2.20	8.78	56.91	1.96	−2.63	0.30
WAL B 34m	34	0.28	0.56	0.49	0.71	2.53	10.49	51.87	1.50	−2.22	0.45
WAL B 36m	36	0.44	0.79	0.51	0.65	3.94	10.53	72.17	2.08	−2.04	0.35
WAL B 38m	38	0.53	0.56	0.52	0.74	3.63	10.97	57.67	1.59	−3.45	0.30
WAL B 40m	40	0.45	0.56	0.45	0.65	5.24	9.48	59.10	1.89	−2.89	0.30
WAL B 42m	42	0.35	0.46	0.52	0.63	4.29	12.31	160.34	3.95	−1.90	0.48
WAL B 44m	44	0.49	0.66	0.60	0.75	4.86	13.54	169.51	3.79	−2.10	0.30
WAL B 46m	46	0.48	0.63	0.52	0.67	4.59	10.53	99.18	2.85	−1.78	0.47
WAL B 48m	48	0.42	0.35	0.51	0.84	4.11	8.44	80.21	2.88	−2.50	0.30
WAL B 50m	50	0.62	0.64	0.58	0.66	3.70	11.84	171.00	4.38	−2.44	0.30
WAL B 52m	52	0.46	0.59	0.49	0.69	2.61	10.38	122.84	3.59	−2.63	0.30
WAL B 54m	54	0.52	0.66	0.51	0.62	1.92	10.56	52.77	1.51	−2.19	0.31
WAL B 56m	56	0.47	0.26	0.49	0.66	1.74	10.12	37.04	1.11	−2.53	0.30
WAL B 58m	58	0.67	0.57	0.56	0.59	5.11	13.17	98.31	2.26	−2.03	0.41
WAL B 60m	60	0.52	0.75	0.45	0.72	1.74	11.31	60.14	1.61	−2.79	0.48
WAL B 62m	62	0.69	0.71	0.47	0.80	2.22	10.73	52.65	1.49	−2.74	0.30
WAL B 64m	64	0.41	0.51	0.48	0.67	6.09	12.51	126.97	3.08	−2.66	0.30
WAL B 66m	66	0.59	0.61	0.48	0.76	3.53	13.83	94.99	2.08	−2.63	0.34
WAL B 68m	68	0.68	0.57	0.65	0.82	2.40	14.29	125.87	2.67	−1.68	0.30
WAL B 70m	70	0.94	0.80	0.58	0.67	3.01	15.73	105.89	2.04	−2.57	0.41
WAL B 72m	72	0.43	0.39	0.34	0.69	1.14	10.66	27.61	0.78	−1.63	0.44

^a^ Normalization based on Algeo and Maynard (2004).

Trace metal data generally records enriched values, which primarily align with previous studies (Hammer et al. 2019). Trace metal concentrations of each sample were compared to corresponding Al concentrations and converted to enrichment factors (Table 2, Figure 2). Manganese concentrations are generally higher near the bottom of the section, up to ~1200 ppm (Mn_EF_ = 2.5), and rapidly decrease around the SSB to ~500 ppm (Mn_EF_ = 0.7), then slowly fall to ~400 ppm up through the remainder of the section. All other noted trace metals are greater than PAAS (Mclennan 2001; Taylor and Mclennan 1985). Vanadium concentrations begin lower (~150 ppm, V_EF_ = 1.3) with minor variability (< 100 ppm deviation, < 0.2 EF) until ~15 m above the base of the Vendomdalen Member, where there are two points with elevated (> 300 ppm, V_EF_ = 3.0) values before returning to a baseline of ~200 ppm (V_EF_ = 1.6). Another extended increase in concentration occurs between 30 m and 55 m to similarly high values before returning. For Mo concentrations, there are slightly elevated values before the SSB (~40 ppm, Mo_EF_ = 50.0), which decrease at the SSB to ~20 ppm (EF = 25.0). Above 15 m, there is substantially increased variability in Mo concentrations, reaching as high as 140 ppm (Mo_EF_ = 170.0) but generally at a baseline of 50 ppm again (Mo_EF_ = 60.0). Uranium concentrations are relatively static, remaining close to 20 ppm (EF = 10.0) at the bottom of the section and slightly increasing through the Vendomdalen Member up section up to 30 ppm (EF = 15.0).

The Smithian‐Spathian Tl isotopic ratios reported here are all less negative than modern oxic seawater (−6.0) (Owens et al. 2017). The oldest portion of the section (Smithian) as reported here maintains isotopic values around −4.0 (average of −3.9) before the SSB. There is a slight positive shift through the Lusitaniadalen Member up to −3.5 before a rapid shift to ~ −2.5 at the SSB. There is some variability of the isotopic values in the Vendomdalen Member, transitioning between −4.0 and −1.7 (average of −2.6), but overall, there is a general trend from the SSB upward toward less negative values.

## 4 Discussion

### 4.1 Local Redox of the Vikinghøgda Formation at the Wallenbergfjellet Section

The Fe_HR_/Fe_T_ ratios, with values all greater than 0.22, indicate locally anoxic conditions, though there might be intermittent intervals that are less reducing considering fluctuations in this ratio, especially with a few samples being below 0.38 (Raiswell et al. 2018). This agrees with other work showing recurrent anoxic conditions at the Smith Creek section in a separate part of the Boreal Ocean based on pyrite framboid distribution and Mo concentrations (Grasby et al. 2013). About half of the samples (26/48) even reach the possibly euxinic threshold of Fe_py_/Fe_HR_ (Benkovitz et al. 2020), though this is significantly less if a more conservative possibly euxinic threshold (0.7) is used (Poulton and Canfield 2005). This suggests a primarily anoxic but non‐euxinic deposition for the entire section with potentially sulfide‐rich porewaters (Hardisty et al. 2018). The more sulfidic conditions occur at and after the SSB, where there is a rapid increase in Fe_py_/Fe_HR_ from 0.3 to 0.7, possibly indicating a major local expansion of reducing conditions starting at the base of the Spathian at this locality that is maintained through much of the remainder of the section.

The redox‐sensitive trace metals primarily confirm the Fe speciation interpretations. The Mn concentrations of the sediments are generally higher in the Smithian, with 2 of the samples greater than PAAS, which might indicate more oxic (less anoxic) conditions as Mn tends to be enriched from the formation and burial of Mn oxides under a more oxic water column (Algeo and Maynard 2004). The values then drop well below PAAS and remain low for the remainder of the section, showing a notable decrease in Mn content, likely oxides, in the sediments compared to the average Smithian values at this section. This Mn trend is similar to the general trends in Fe speciation, which show lower ratios earlier during the Smithian and higher ratios in the Spathian. Both Fe speciation and Mn proxies mirror the rapid local decrease in benthic fauna, confirming the impact of more reducing local conditions on the environment (Blattmann et al. 2024; Hansen et al. 2024). These Mn concentrations also indicate minimal impact on the Tl isotopic composition from the incorporation of Mn oxides (Nielsen et al. 2011).

Throughout the section, V, Mo, and U are all enriched, though V in particular is only slightly enriched (< 2 × PAAS) for much of the section (Figure 2). Enrichment of these elements indicates locally anoxic conditions throughout this interval. Of note is an interval of enrichment in both V and Mo around 40 m to 50 m (V_EF_ > 4, Mo_EF_ > 80) that could be interpreted as a major episode of more intense locally reducing conditions sequestering more redox‐sensitive elements, all within the window of raised Fe_py_/Fe_HR_. Additionally, consistently elevated U concentration (~10 × PAAS) along with various spikes in V and Mo concentrations throughout the Spathian suggest generally anoxic conditions in the Boreal Ocean.

Several previous studies have shown variable redox conditions within this region but generally confirm that there were predominantly widespread anoxic depositional conditions (Blattmann et al. 2024; Grasby et al. 2013, 2016; Hammer et al. 2019). This is most notable with enhanced Mo burial in the adjacent Sverdrup Basin, indicative of the anoxic marine conditions described at that site (Grasby et al. 2013, 2016). However, the fluctuations in Mo, as well as V, do not closely match the large‐scale changes in sea level at this time (Blattmann et al. 2024; Embry 1988; Hansen et al. 2024) or global changes in redox conditions, which primarily fluctuate across the SSB rather than during the entire Spathian (Blattmann et al. 2024; Grasby et al. 2013; Hammer et al. 2019; Zhang et al. 2019; Zhao et al. 2020). It is also possible that the close proximity of the Boreal Ocean to the Siberian Traps (Figure 1) may have allowed for input of volcanically sourced trace elements into this specific basin (Grasby et al. 2016; Hammer et al. 2019), causing temporal anomalies in elemental concentrations due to different eruptive phases. However, elemental enrichment similarity to concentrations indicates that these increases are not likely due to an influx of source material as aluminum, tracking source material, is relatively invariable. There are similarly timed changes in mercury (Hg) concentrations (Grasby et al. 2016; Hammer et al. 2019), an indicator of volcanic activity. However, correcting these values to total organic carbon—as Hg can preferentially be adsorbed to organic matter—shows minimal change in the Spathian Hg contents and low enough values to indicate a drastic decrease in Siberian Trap volcanism (Hammer et al. 2019). It is possible that intense weathering of nearby nonvolcanic material can also periodically increase these trace elemental abundances in marine settings, but the close similarity between concentrations and enrichment factors, which are used to account for changing sedimentation rates due to erosion and deposition, indicates that significant changes in weathering are unlikely a primary cause. Therefore, the primary cause of fluctuations in Mo and V throughout the Spathian is unlikely to be volcanically driven, and thus likely driven by redox conditions.

### 4.2 Global Redox Conditions Around the Smithian‐Spathian Boundary

In specific scenarios, V, U, and Mo concentrations can provide information on global redox conditions through marine trace‐metal inventories. These elemental data are elevated throughout the section but often not highly enriched compared to PAAS and expected enrichments under somewhat anoxic conditions (Algeo and Maynard 2004; Lyons et al. 2009; Mclennan 2001; Taylor and Mclennan 1985). We see no difference in trends based on direct concentrations or other normalization methods. Previous work has shown that, though local enrichments of these trace metals is common under anoxic conditions (Algeo and Maynard 2004; Lyons et al. 2009), they ultimately depend on a globally large reservoir of these elements in seawater (Owens et al. 2016; Reinhard et al. 2013). As global anoxic conditions become more widespread or intense, V, U, and Mo would be more efficiently removed from seawater until the seawater content is removed at a greater rate than renewal by the various source fluxes. At this point, any anoxic basins would not have sufficient dissolved elements to enrich sediments, causing a depleted sedimentary value (Owens et al. 2016; Reinhard et al. 2013). The Wallenbergfjellet trace metal concentrations are still enriched compared to PAAS, suggesting a sufficient global reservoir of all of these elements must be present to allow for such local enrichments. There is the possibility of globally anoxic conditions, albiet not sufficiently widespread or reducing enough (i.e., euxinic) at a global scale to cause significant reservoir drawdowns of these trace metals (Lyons et al. 2009; Owens et al. 2016; Reinhard et al. 2013).

For further insights, Tl isotopes are utilized to provide a mechanism to interpret the global redox conditions as related to Mn oxide burial. There is little indication that the Tl isotopic values are related to any local mineralogic or geochemical controls on the sediments beyond recording the overlying water column chemistry (see Supplemental Discussion and Figure S1). Local proxy data for bottom water anoxic conditions indicate sedimentary Tl isotopes reported here should represent overlying Early Triassic seawater (Fan, Nielsen, et al. 2020; Newby et al. 2025; Owens et al. 2017; Wang et al. 2022). The base of this section, below the SSB (< 0 m) has Tl isotopic values around −4.0, which is less negative than modern oxic seawater values (Owens et al. 2017) but also not as positive as source values (Nielsen et al. 2017; Owens 2019), with source‐like values being found during the earliest Triassic following the EPME and indicative of limited global Mn oxide burial due to widespread anoxia (Newby et al. 2021). Therefore, the late Smithian values of −4.0 indicate a decrease in the spatial extent of reducing conditions from the earliest Triassic but not to the levels of modern primarily oxic oceans. This is broadly consistent with many other studies of the Early Triassic that have found persistent anoxic conditions throughout the Early Triassic based on Fe speciation, pyrite framboid data, trace metal concentrations, and uranium isotopes (Grasby et al. 2013; Song et al. 2012, 2019; Wu et al. 2024; Zhao et al. 2020).

At the SSB, there is a rapid perturbation toward less negative Tl isotopic values, indicating a rapid expansion of anoxia at potentially a global scale, though a potential latest Smithian hiatus at Wallenbergfjellet may partly account for the rapid changes (Hansen et al. 2018). Like other Phanerozoic anoxic events, such as the Late Ordovician mass extinction, Silurian Lau event, Jurassic Toarcian Oceanic Anoxic Event, and Cretaceous Anoxic Event 2, maximum extent of anoxia is reached in a stepwise fashion from non‐modern (not −6.0) values (Bowman et al. 2019; Kozik et al. 2022; Ostrander et al. 2017; Them et al. 2018). These anoxic events often coincide with minor, or even major, marine extinctions that can be attributed to the small but rapid oceanic redox transitions (Bond and Grasby 2017; Bowman et al. 2019; Jenkyns 2010; Kozik et al. 2022), matching the SSB rather than the larger change noted for the EPME (Newby et al. 2021).

Following the SSB, there is some variability in the Tl isotopic values, transitioning between −2.0 and −4.0, though most values are primarily close to −2.0, similar to several other anoxic events studied using Tl isotopes (Bowman et al. 2019; Kozik et al. 2022; Newby et al. 2021; Ostrander et al. 2017; Them et al. 2018). During the Spathian, Tl isotopes briefly fluctuate from −2.0 to −3.5 and −4.0 at ~15 and ~40 m, respectively, though not contemporaneous with trace metal variance, which may indicate global‐level oceanic redox variance in extent or intensity. Global redox fluctuations would then affect the baseline concentrations and isotopes of all the geochemical signatures. There is a lack of evidence that any of these are associated with other events throughout the Spathian, such as sea level change or climate disturbance (Grasby et al. 2013, 2016; Song et al. 2013; Zhang, Romaniello, et al. 2018; Zhang et al. 2019; Zhao et al. 2020). As Tl has both a short residence time and responds to the initial changes in oxygen content more than most global redox proxies, it may be that transient reoxygenations are recorded over this extensive interval that would be difficult to note in other proxies which are sensitive to changes further down the redox ladder. There may be some relation to biotic diversity, as the Wallenbergfjellet section shows increases in biota, especially bivalves, at certain intervals of more oxic conditions in Tl isotopes, especially below 0 m and around both 20 and 39 m (Hansen et al. 2024). However, redox conditions do not return to oxic conditions by the end of the Early Triassic.

### 4.3 Potential Restriction of the Boreal Sea

Along with the latest Smithian hiatus, there are some uncertainties to this global interpretation that must be addressed through other means due to the Boreal Ocean being more polar and somewhat separated from the main ocean basins (i.e., requiring longer mixing times) during this period compared to other studied sections (Foster et al. 2015; Galfetti, Bucher, et al. 2007; Galfetti, Hochuli, et al. 2007; Song et al. 2019; Zhang, Romaniello, et al. 2018; Zhao et al. 2020). First, the Fe_T_/Al ratios can potentially be used to determine generally if a basin is restricted through the formation of a strong Fe shuttle causing local Fe enrichment (above 0.55), which is most noted in several restricted settings (Lyons et al. 2009; Raiswell et al. 2018; Severmann et al. 2008), though may occur in open marine settings to a lesser degree (Scholz et al. 2014). The majority (> 75%) of samples were within one standard deviation of average modern oxic sediments in Fe_T_/Al (0.44–0.66), and the few that were beyond this range showed only small deviations (Figure 2). Thus, these values indicate greater similarity to modern oxygen minimum zone values than below these zones (Scholz et al. 2014) or below a highly restricted water column (Lyons et al. 2009; Severmann et al. 2008).

**FIGURE 3:**
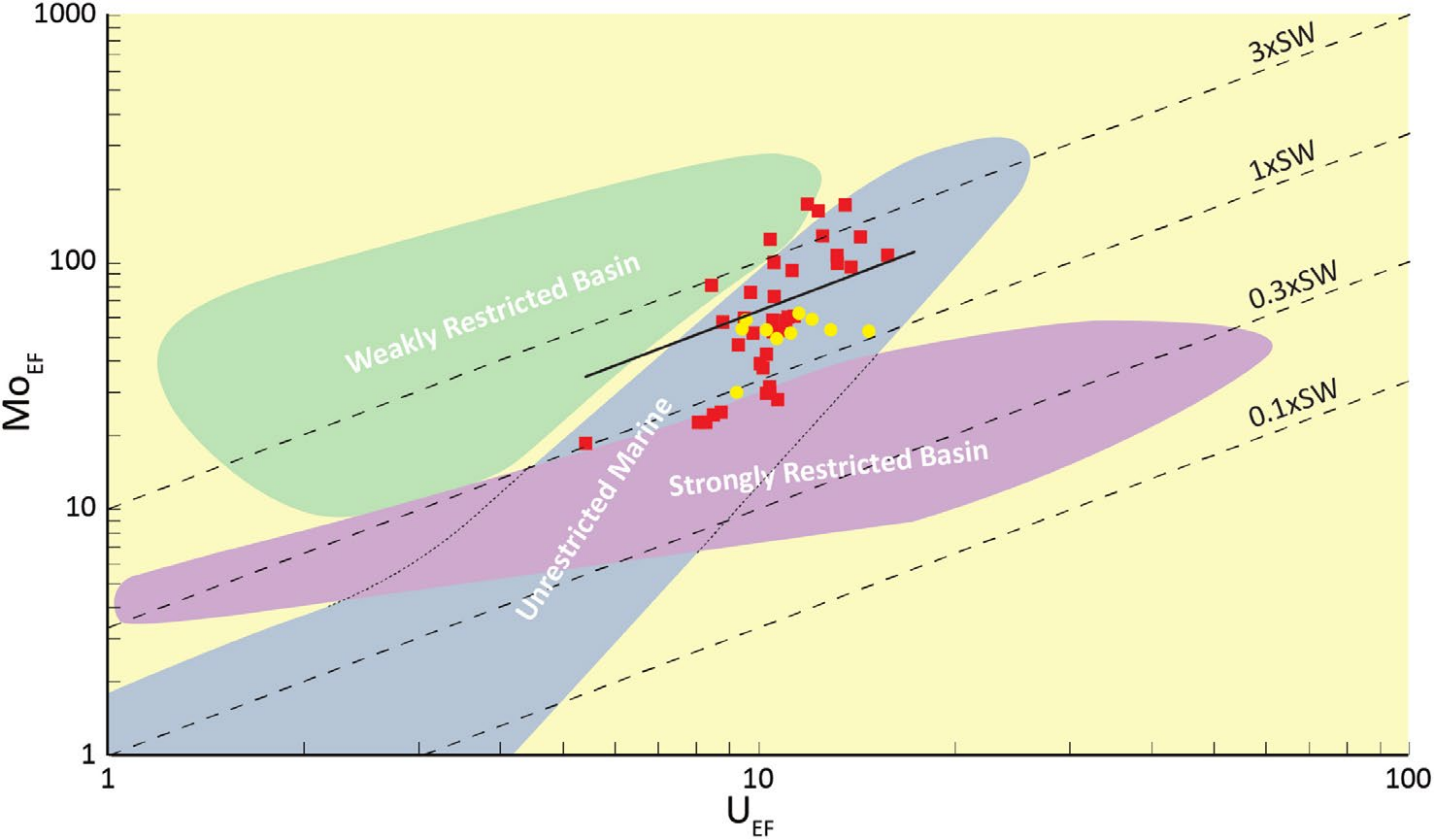
Mo_EF_ versus U_EF_ for Wallenbergfjellet samples. Seawater ratio (1 × SW) as well as multiples and fractions of seawater (3.0 × SW, 0.3 × SW, and 0.1 × SW) are included (dashed lines) as in previous studies of redox evolution of a location (Algeo and Tribovillard 2009). Approximate fields for different local redox evolutions, along with their naming conventions, are based on data and descriptions from Algeo and Tribovillard (2009). Modern environments considered unrestricted marine include the Peru shelf, Mexico margin, and central to south California margin and basins; weakly restricted basins include Cariaco and Orca basins, and strongly restricted basin such as the Black Sea. Yellow circles for Smithian samples and red squares for Spathian samples. Linear regression of all samples across all data is shown as a solid black line. Note: axes are logarithmic.

More notably, U_EF_ versus Mo_EF_ plots (Figure 3) were utilized to compare modern reducing basins to ancient sediments and determine the processes that have induced these low‐oxygen conditions (Algeo and Tribovillard 2009). The overall clustering of the data, plotting along and above the 1 × SW values, is distinct from most of the fields that are indicative of strongly restricted basin development, but may be similar to modern Mexico and Peru margin sediments, unrestricted marine settings (Algeo and Tribovillard 2009). Notably, the clustering of data suggests limited change in basin hydrography across the studied interval, even when separating Smithian and Spathian datasets to check temporal change (Algeo and Tribovillard 2009).

Additionally, there are several open‐marine faunal groups (e.g., ammonoids and conodonts) that can be found at Wallenbergfjellet before and after the SSB (Hammer et al. 2019; Hansen et al. 2018; Leu et al. 2024), as well as a similar organic carbon isotope excursion at this site compared to the global carbonate carbon isotope excursion found across the SSB and into the Spathian (Blattmann et al. 2024; Hammer et al. 2019), both indicating global ocean connectivity. All of these methods indicate Tl isotopes found at Wallenbergfjellet are likely representative of the global ocean values throughout the study interval.

However, as noted, the rapid shift in Tl isotopes co‐occurs with changes in Mn content, Fe_py_/Fe_HR_ ratios, and a hiatus in deposition (Blattmann et al. 2024; Hansen et al. 2018), with the hiatus indicating that the geochemical signatures may be altered by a restriction of the regional ocean basin from the wider ocean input (Wu et al. 2024) causing a change in local redox for the local proxies and a transition to source‐dominated Tl isotopes. This would also be expected to alter the other various trace metals to a notable degree, eventually causing depletion of V, Mo, and U due to basin‐wide burial, similar to what is documented in the modern‐day Black Sea (Lyons et al. 2009), but unlike the enrichments shown here in the Early Triassic (Figure 2).

Even with various potential indications of open marine conditions, there is still a possibility that the Boreal Ocean or the specific basin that the Wallenbergfjellet section resided in was sufficiently restricted to affect the Tl isotopic values without global change in marine redox. Biostratigraphic and sequence stratigraphic evidence suggests the latest Smithian interval experienced a sea‐level drop, to the point of non‐deposition and/or erosion, associated with cooling during this substage (Hammer et al. 2019; Hansen et al. 2024; Leu et al. 2024; Song et al. 2019; Wu et al. 2024). This could drive the Tl isotopic values toward source values as the lack of regional Mn oxides limits fractionation of basinal seawater while continual riverine input shifts isotopic values closer to source values. This would be similar to the modern‐day Black Sea's aberrant isotopic values of −2.0, that are very different from open ocean values near −6.0 (Owens et al. 2017). Due to the relatively short residence time of Tl in seawater, this isotope system requires consistent mixing with global seawater to not be controlled by regional sources to the basin. As Tl isotopes shift from more negative values to less negative, source‐like values at the SSB, where there is a known hiatus (Hansen et al. 2018, 2024), it is very conceivable that a lowering of sea‐level caused a restriction of the Boreal Sea that allowed for rapid regional deoxygenation across the SSB (Wu et al. 2024). Nevertheless, this restriction scenario is unlikely as there are several perturbations in the Tl isotopic value above the SSB, and most are not associated with changes in sea‐level or temperature (Song et al. 2019; Sun et al. 2012; Wignall et al. 2016; Wu et al. 2024; Zhang et al. 2019) nor are they associated with notable changes in other global proxies (Zhang, Algeo, et al. 2018; Zhang, Romaniello, et al. 2018; Zhao et al. 2020). Further discussion assumes global connectivity of the Boreal Sea to the wider ocean, but if restriction were the dominant control on the Tl isotopic composition of these sediments, these interpretations would be relegated to the Boreal Sea rather than the global ocean.

It is possible that a degree of restriction occurred similar to Cariaco Basin, a basin sufficiently restricted from the global ocean to become highly stratified with euxinia present but still with enough connection to the open ocean to record global seawater Tl isotopic values (Chen et al. 2021; Owens et al. 2017). This may explain the transition of some local redox proxies, such as Fe speciation and Mn concentrations, to a more anoxic state at the SSB while other local proxies, namely the remaining trace metal concentrations, maintain little change across the SSB as some connection to the open ocean remained. Under such a scenario, Tl isotopes are likely to work as they do in the modern‐day Cariaco Basin, where they record open ocean seawater values rather than restricted marine conditions like those found in the Black Sea (Owens et al. 2017).

### 4.4 Changes in Redox Across the Latest Permian to Early Triassic

**FIGURE 4:**
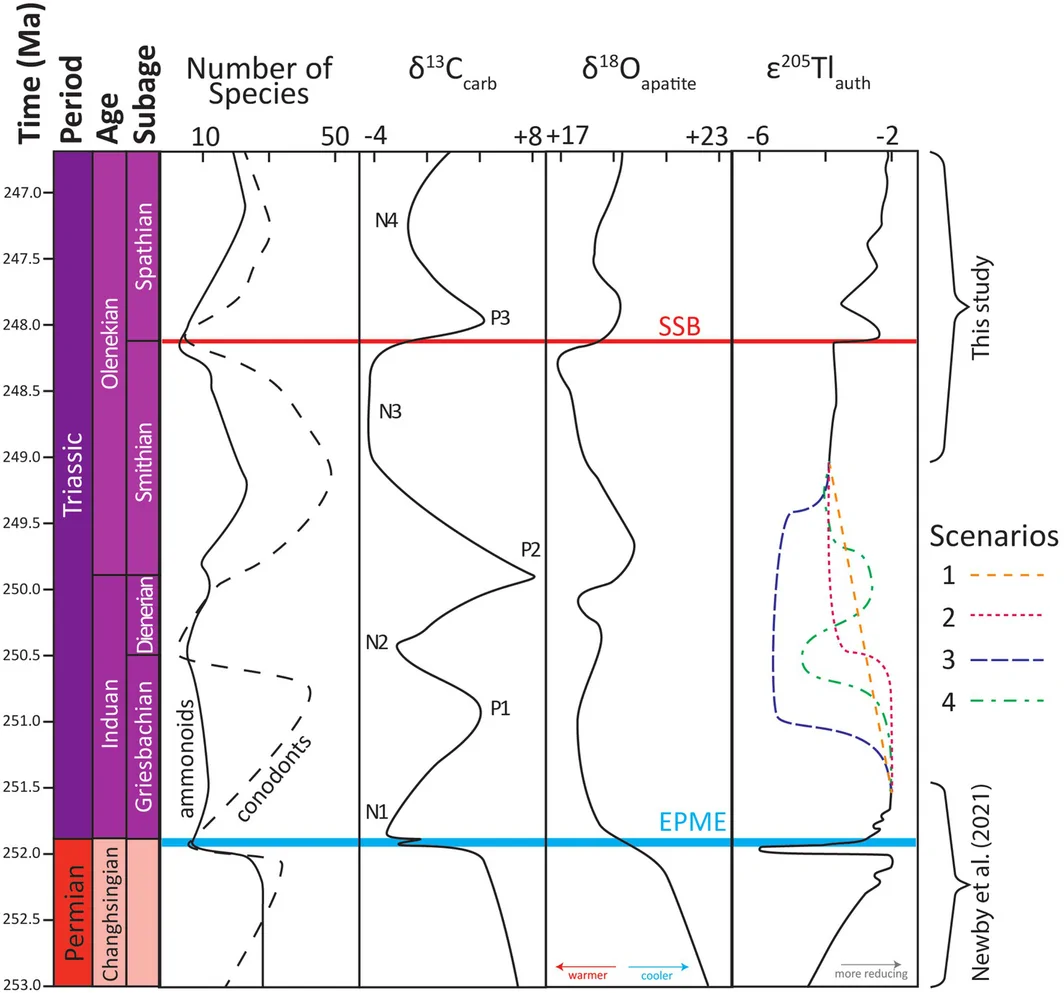
Idealized trends and isotopes of the Early Triassic. Generalized diversity record and carbon, oxygen, and thallium isotope curves of the latest Permian and Early Triassic for simplified interpretation of Early Triassic changes. Chronology of the Early Triassic based on Ogg et al. (2020), though Dai et al. (2021) indicates some potential changes in time of specific events. Diversity curves are simplified ammonoid (solid line) diversity (Brayard et al. 2006; Fan, Shen, et al. 2020; Kiessling et al. 2018; Stanley 2009) and conodont (dashed line) diversity (Fan, Shen, et al. 2020; Hansen et al. 2024; Martínez‐Pérez et al. 2014; Orchard 2007; Stanley 2009) across the latest Permian through the Early Triassic. Carbon data are idealized from several previous studies (Payne et al. 2004; Song et al. 2013; Sun et al. 2012). Carbon isotope excursion naming schemes (N1–N3 and P1–P3) follow previous work (Song et al. 2013), with Ns for negative carbon excursions and Ps for positive carbon excursions. Oxygen isotopes are from a moving average of a previous global compilation (Sun et al. 2012) supplemented with detailed Tethyan work (Romano et al. 2013) and altered timing of climatic changes (Song et al. 2019). Thallium isotopes come from an idealized curve presented from the Panthallasic Ocean across the EPME (Newby et al. 2021), matching more recent data from the Paleotethys Ocean (Wang et al. 2026), and are combined with a moving average of this study. Colored dashed lines in the Tl curve represent potential scenarios for the gap in data between the two studies (see Supplemental Discussion). Exact timing of changes in Tl isotopes across this gap is not completely certain. The EPME is represented by a horizontal blue line and the SSB by a horizontal red line.

These data can help to expand on the ocean redox state from the latest Permian through the Early Triassic (Figure 4), similar to work with other global redox proxies like U isotopes (Zhang, Algeo, et al. 2018; Zhang, Romaniello, et al. 2018; Zhao et al. 2020). Leading up to the Permian–Triassic boundary, there exists a gradual positive trend in isotopic values from −4.0 to −2.0 (Newby et al. 2021) over the span of a million years (Algeo, Kuwahara, et al. 2011), followed by large transient fluctuations in redox at the EPME, briefly returning to oxic values before becoming highly anoxic, possibly inducing or exacerbating the extinction (Newby et al. 2021). This occurs approximately in conjunction with a major negative carbon excursion that marks the EPME, potentially indicating the global biogeochemical changes recorded in carbon isotopes may correlate with changes in Tl isotopes. These anoxic conditions in the oceans remain well into the Griesbachian of the Early Triassic (Newby et al. 2021), though the duration of anoxia is uncertain due to limited time constraints at the stratigraphically youngest section (Schoepfer et al. 2013).

This leaves a potentially large gap (~1–3 Myr) before the start of this study in the Smithian (Ogg et al. 2020), where values have shifted from −2.0 to be closer to −4.0, with the remainder of the Early Triassic already described. It is possible, due to the time scales being invoked, that alteration of oceanic crust could become a viable source of change in seawater Tl isotopic values (Nielsen et al. 2017), but this implies an unlikely major change in global subduction rates considering the continued presence of Pangea and the minor fractionation this sink has from seawater (Prytulak et al. 2013; Shu et al. 2022). More likely, there are several redox‐related possibilities as to what occurred during this gap (Figure 4): (1) a steady relative decrease in anoxic bottom waters, (2) a rapid transition from −2.0 to −4.0 during the later Griesbachian, Dienerian, or early Smithian, (3) a return to modern oxic values in the late Griesbachian or Dienerian before returning to anoxic conditions in the Smithian, or (4) multiple swings between more and less oxic conditions throughout the Early Triassic, likely in conjunction with changes in carbon isotopes (Supplemental Discussion). The most likely scenarios are steady decrease in anoxic bottom waters as this may imply the smallest overall change in ocean conditions or the high fluctuations in redox conditions to match the carbon isotopes, with the latter being even more likely based on other redox studies indicating variability in Early Triassic marine redox (Grasby et al. 2016; Wu et al. 2024). These likely scenarios mostly imply no long‐term return to oxic conditions this is similar to other proxies that suggest intense reducing conditions, indicating widespread marine anoxia through the majority of the Early Triassic, even if the degree of anoxia is variable (Grasby et al. 2013; Hammer et al. 2019; Song et al. 2013; Wignall et al. 2016; Zhao et al. 2020). However, the gap in data limits the precise interpretation of geochemical changes prior to the Smithian.

### 4.5 Implications for the Smithian‐Spathian Extinction

The SSB is noted for the extinction of multiple groups across the boundary after recently recovering from the EPME. Though the precise timing of this extinction has been debated in relation to the available biostratigraphy and fossil abundance, determinations of stratigraphic boundaries, chemostratigraphic correlations, and environmental indicators, this work assumes an extinction at the SSB. Recent work following the most recent timescale update of the Triassic (Ogg et al. 2020) shows that the extinction may actually occur at the limitedly constrained middle to late Smithian boundary rather than at the later SSB based on regional fossil assemblages and biostratigraphy (Dai et al. 2021). However, some missing material from around the SSB in parts of the Boreal Ocean, such as at Wallenbergfjellet, makes it hard to determine if this difference is recognizable (Hansen et al. 2018; Leu et al. 2024). As current regional evidence indicates an extinction at the boundary that works in combination with changes in carbon isotopic changes (Hammer et al. 2019; Hansen et al. 2018, 2024; Leu et al. 2024), we maintain the extinction at the SSB rather than slightly earlier in further discussion, though it may be earlier and just not sufficiently diagnosed in this region.

Within the Boreal Ocean, there are changes in vertebrate, ammonoid, and conodont faunas, and even pollen spectra from nearby terrestrial sources (Hansen et al. 2018, 2024; Leu et al. 2024), all showing the local effects of extinction. On a global scale, this crisis is most noted in ammonoids and conodonts (Figure 4) that experienced rapid loss both at the EPME and the SSB (Brayard et al. 2006; Fan, Shen, et al. 2020; Hansen et al. 2024; Kiessling et al. 2018; Martínez‐Pérez et al. 2014; Orchard 2007; Stanley 2009), though various other fauna, such as foraminifera, radiolarians, bivalves, and vertebrates, all exhibited some degree of loss across the interval (Fan, Shen, et al. 2020; Galfetti, Hochuli, et al. 2007; Hansen et al. 2024; Leu et al. 2024; O'Dogherty et al. 2009; Song et al. 2011; Sun et al. 2012). There is a strong impact on the various nektonic groups across this interval, namely ammonoids and conodonts (Fan, Shen, et al. 2020; Galfetti, Hochuli, et al. 2007; Hansen et al. 2018, 2024; Leu et al. 2024; Stanley 2009; Sun et al. 2012; Zhang et al. 2019), indicating that one of the notable kill mechanisms may have been anoxia persisting in the water column to induce extinction. Even without species loss, a size reduction in several ammonoids shows the environmental impact on large nektonic creatures (Hansen et al. 2024). More compelling is the extinction of several benthic groups, notably certain bivalves, though there is loss in some gastropods as well (Dai et al. 2021; Galfetti, Hochuli, et al. 2007; Hansen et al. 2024; Leu et al. 2024), which matches the limitation of global Mn oxide formation and burial in the sediments due to bottom water deoxygenation. Additionally, the change in latitudinal diversity gradients (Brayard et al. 2006; Galfetti, Bucher, et al. 2007; Galfetti, Hochuli, et al. 2007) indicates that changes in anoxia may be unequal globally or be temporally variable, limiting the degree of extinction compared to larger events such as the EPME. This is followed by limited recovery in the early Spathian, both in terms of species occurrence and small size of generally large organisms (Hansen et al. 2024).

As mentioned, the SSB represents the cooling phase that followed the climate maximum surrounding the EPME (Figure 4), which is both a potential kill mechanism and a causal mechanism for the increased anoxia, though the latter is hard to reconcile as most anoxic events are associated with warming intervals (Bond and Grasby 2017; Jenkyns 2010). It is possible that this cooling phase induced major recirculation of the oceans that brought sufficient nutrients to the surface to invigorate primary productivity and inducing a minor anoxic event. This has been postulated as a potential source of widespread anoxia for the SSB (Song et al. 2019) and aligns with the sudden positive perturbation in Tl isotopes and indicators of increased nutrient fluxes (Blattmann et al. 2024). Even with this possibility, the cause of cooling is uncertain, with many speculating it is either related to a phase of Siberian Traps eruption that may have released aerosols and SO_2_ that could induce cooling (Black et al. 2018; Grasby et al. 2016; Song et al. 2019) or a ceasing of eruptive activity from the Siberian Traps that caused cooling through lack of additional CO_2_ release (Hammer et al. 2019; Song et al. 2019). As the later phases of Siberian Trap activity in the Early Triassic are not well dated in comparison to the timeframe surrounding the EPME (Burgess et al. 2017), it is difficult to assess how these might relate or even if the Siberian Traps are implicated at all. The Hg concentration and ratios both suggest (Grasby et al. 2016) and oppose (Hammer et al. 2019) potential volcanism in the region around the SSB, further complicating interpretations. Additionally, changes in ocean circulation could have been induced by ongoing changes in plate configurations, allowing for invigoration of upwelling or movement of oxygen minimum zones that can affect the global distribution and abundance of marine anoxia (Song et al. 2019). However, the general distribution of tectonic plates is thought to have been relatively unchanged since the EPME and even before due to the formation of Pangaea, making it unlikely to be a primary cause of the SSB extinction or diminished Early Triassic recovery as a whole.

The variability in the redox state of the ocean could hinder recovery as organisms were unable to adapt to the significantly fluctuating redox conditions. The more intense anoxic conditions found in the earliest Spathian and rapid transition to such a state across the SSB would indicate that this was a likely causal mechanism for the extinction, similar to the rapid changes at the EPME inducing extinction (Newby et al. 2021). Additionally, the overall generally reducing but somewhat variable ocean conditions that occurred later in the Spathian would likely have played a role in the slow recovery during the entirety of the Early Triassic. There are multiple potential scenarios as to the development and degree of anoxia (Figure 4) across the Early Triassic, but most indicate that anoxia would persist through much of this interval, matching other proxies across this time and explaining the slow recovery (Grasby et al. 2013; Hammer et al. 2019; Song et al. 2012, 2013; Wignall et al. 2016; Zhao et al. 2020). Several of these records show variability in marine redox (see Scenario 4 in Figure 4) exacerbating the biotic recovery (Grasby et al. 2013; Song et al. 2012; Wu et al. 2024). This scenario most closely aligns with changes in biodiversity across this interval, primarily within the conodont and ammonoid records (Stanley 2009; Sun et al. 2012). Thus, variable redox conditions were likely contributors to slow Early Triassic recovery, and the most extreme fluctuations in redox were related to the EPME and SSB crises where multiple groups of organisms were affected.

## 5 Conclusions

The Early Triassic is considered an interval of slow recovery following the massive EPME. This is momentarily interrupted by the SSB minor extinction event. All of these documented changes in the biotic records are considered to be induced by major marine redox changes. Therefore, a suite of redox sensitive metal proxies was employed on the Svalbard samples to better constrain regional and global redox conditions. Here, Fe speciation and trace metal concentrations confirm other paleoredox studies within this basin that there is generally an anoxic state within the Boreal Ocean, with intervals of time that became even more reducing (i.e., sulfidic conditions). There is some variability in the regional redox conditions as trace metal concentrations fluctuate over the interval, but reducing bottom water conditions persist throughout. Additionally, Tl isotopes likely show an increase in globally reducing conditions throughout the Smithian‐Spathian boundary interval, although further studies outside of the Boreal Ocean are needed to confirm our Tl isotope records are global in nature. A sudden shift in Tl isotopes to less negative values at the SSB shows that these anoxic conditions became more widespread at this boundary, likely inducing the small extinction event that is recorded at this interval. Taken in conjunction with the Tl isotopes for the EPME, there appears to be widespread anoxia throughout the Early Triassic that, though potentially fluctuating, doesn't dissipate by the end of the epoch. This widespread anoxia, and sudden shifts to more intense anoxic conditions, are likely contributing factors to the long‐term recovery of the Early Triassic fauna.

## Funding

S.M.N funded by FSU EOAS Winchester fund. J.D.O funded through the NASA Exobiology program (NNX16AJ60G and 80NSSC18K1532) and the Sloan Research Foundation (FG‐2020‐13552). Ø.H. funded through the Swiss SNF (project 200020‐160055). The National High Magnetic Field Laboratory in Tallahassee, FL is supported by the National Science Foundation Cooperative Agreement (No. DMR‐1644779) and the State of Florida.

## Conflicts of Interest

The authors declare no conflicts of interest.

## Supporting information


**Table S1:** Fe speciation duplicate values, bolding duplicated values.
**Table S2:** Trace metal geostandard values, bolding reported values.
**Figure S1:** Various geochemical components versus thallium isotopes.

## Data Availability

All data is included in the provided tables of the main text and Supporting Information.
