# Supplementary material for: Thallium Isotopes Across the Smithian‐Spathian Boundary: Evidence for Anoxia Throughout Most of the Early Triassic
Source: Geobiology. 2026 Sep 2;24(5):e70062. doi: 10.1111/gbi.70062 (PMC13539283; doi:10.1111/gbi.70062)
Supplement: Supplementary file 1 [file GBI-24-e70062-s001.docx]

**S1. Supplemental Discussion:**

**S1.1 Sedimentary controls on thallium isotopes**

There are several potential controls on Tl isotopes in the sediments that would preclude its capability as a redox proxy (Fig. S1). A recent study on Tl isotopic compositions of a ferruginous lake with high organic carbon content indicates there may be a negative fractionation of Tl adsorbed to organic matter (Ostrander et al., 2025). They show a relationship between total organic carbon (TOC) and ε^205^Tl_auth_, with higher TOC correlating to lower Tl isotopic values. This does require extremely high TOC (~16 Wt%) to induce a small Tl isotopic fractionation (~-1.0 ε-units). However, our data indicates an inverse relationship, if one does exist considering the low correlation (R^2^ = 0.33), with higher TOC samples (Hammer et al., 2019; Hansen et al., 2018) having more positive Tl isotopic compositions. Therefore, the organic content of these samples is likely not causing an isotopic fractionation of the Tl isotopes, and they probably are not the primary authigenic phase in which Tl has incorporated.

Another major phase to consider is the potential for Mn oxides to be incorporated into the signal (Nielsen et al., 2013; Rehkämper & Nielsen, 2004). This has been discussed in text, but we further look into the potential relationship by cross-plotting the data. This would expect the highest Mn values to also have the most positive Tl isotopic values, but, again, an inverse relationship exists. The low correlation (R^2^ = 0.23) further represents little control on the sedimentary isotopic values from inclusion of Mn oxides. Instead, the increased Mn concentrations in the Smithian may represent alternative Mn minerals, which would better compare to the other data for the Smithian.

The last notable phase for Tl to be incorporated into is in pyrite (Nielsen et al., 2011; Owens et al., 2017), which the Fe_py_/Fe_HR_ ratio and Mo_EF_ may both represent due to the former being a representation for degree of pyrite formation (Poulton & Canfeld, 2011) and the latter a trace metal tracer closely tied to sulfidic content of sediments (Algeo & Maynard, 2004). Neither of these have close correlations to the Tl isotopic composition (R^2^ = 0.04 for Fe_py_/Fe_HR_; R^2^ = 0.15 for Mo_EF_). However, as pyrites are assumed to incorporate overlying seawater Tl without fractionation due to quantitative adsorption (Nielsen et al., 2011; Owens et al., 2017), they should not have any correlation to the isotopic composition of their respective Tl isotopes. Therefore, these relationships are to be expected and pyrite on their own are not controlling the isotopic composition of the samples but rather recording the overlying water compositions as expected. There is also no control on the isotopic composition of Tl by the amount of Tl in the sediments (R^2^ = 0.11), as has been shown in several other instances where Tl isotopic values are not related to the concentrations (Chen et al., 2021; Ostrander et al., 2017; Owens et al., 2017; Them et al., 2017).

**S1.2 Scenarios for Early Triassic thallium isotope development**

There are several scenarios that we present for the interval between the earliest Griesbachian Tl isotopic values (Newby et al., 2021) and the current study on the Smithian-Spathian interval. These were necessary due to a large gap in available data for global interpretations of Tl isotopes, which can be estimated to be ~1-3 Myr. The uncertainty in length is due to limited biostratigraphic constraints for the samples in the Griesbachian (Schoepfer et al., 2012, 2013) and Smithian (Hansen et al., 2018, 2024; Leu et al., 2024). With this length of time and Tl’s relatively short residence time (Owens et al., 2017), it is conceivable that the Tl isotopic composition of this interval is highly fluid and may change drastically. However, with the main isotopic control on Tl isotopes across short time intervals being changes in Mn oxide formation and burial rates (Owens, 2019), the primary limiter for changes in Tl isotopes is global changes in marine redox. There are several intervals that show rapid changes in marine redox state, such as the various oceanic anoxic events of the Mesozoic, but these still primarily show a large expansion and then contraction of anoxic conditions across multiple hundreds of thousands to millions of years (Bond & Grasby, 2017; Jenkyns, 2010). Looking more specifically at Tl across several anoxic events, it is evident that the initial change towards expanded anoxic conditions can be relatively quick but the recovery following these events is much slower (Bowman et al., 2019; Kozik et al., 2022; Newby et al., 2021; Ostrander et al., 2017; Them et al., 2018). Therefore, though a potential 3 Myr (Ogg et al., 2020) may have passed in this uncertain interval, it is likely that only a few shifts in Tl isotopes at most may have occurred during the Early Triassic. It should be noted, with this interval being several million years, the entire isotopic change may be controlled by changes in low temperature alteration of oceanic crust, which changes on multi-million-year timescales (Nielsen et al., 2017), though this has yet to be interpreted in deep-time studies. This leaves the four listed scenarios as the most likely possibilities, though these are not definitive as described or other possible scenarios may have been more accurate but were not considered.

Of the various scenarios represented connecting Newby et al. (2021) and this study, scenario 1 is the most straightforward. This represents a linear increase in Mn oxide burial between the Griesbachian and the Smithian. This would represent a relatively slow recovery from the anoxic state of the end-Permian mass extinction (EPME), matching other work for Early Triassic recovery from the widespread global anoxic state of this massive extinction interval (Wignall et al., 2016; F. Zhang et al., 2018; Zhao et al., 2020). A similar alternative is scenario 2, where widespread anoxic values are maintained long after the EPME, then experience a rapid decrease in isotopic value to a state that is similar to what is found in the Spathian of this study. This may align with other studies on Tl isotopes following anoxic events that show little deviation for hundreds of thousands of years past where the notable carbon isotopic excursion and lithologic data indicate a conclusion to these events (Bowman et al., 2019; Ostrander et al., 2017; Them et al., 2018). The exact mechanism for this is unknown but is potentially linked to perpetually anoxic sediments minimizing Mn oxide burial rates, even after reoxygenation of overlying waters (Them et al., 2018). This may not need to be invoked for the Early Triassic, however, as several studies do indicate widespread anoxia following the EPME (Grasby et al., 2013; F. Zhang et al., 2020; Zhao et al., 2020). In general, scenario 1 and 2 are similar in representing a broadly anoxic marine state that becomes less anoxic prior to the Smithian while never returning to modern oxic values for Tl isotopes, though the exact rate of change from Griesbachian is different. This may be pertinent as rapid changes in Tl isotopes, and therefore global marine redox, may be relevant to extinctions no matter if this represents an increase or decrease in anoxia (Kozik et al., 2022; Newby et al., 2021), and as the Smithian-Spathian boundary (SSB) represents the next notable extinction following the EPME (Foster & Sebe, 2017; Galfetti et al., 2007; Song et al., 2019; L. Zhang et al., 2019), it seems likely that rapid change in anoxia, and therefore the relation to Tl isotopic compositions, were not in play during this interval.

Separately, scenario 3 represents a wholly different scenario in which Tl isotopic values return to modern seawater composition, indicating an increase in Mn oxide burial to a similar condition as modern oceans. This would broadly indicate a recovery from the EPME, with a later point, likely around the early Smithian, returning to less oxic conditions. There is some data that suggests more recovery of certain groups between the EPME and SSB that could be related to changes in ocean redox (Song et al., 2019; F. Zhang et al., 2020), however, there is minimal evidence of widespread oxic conditions in the Early Triassic among the various proxies employed (Grasby et al., 2013; F. Zhang et al., 2018; Zhao et al., 2020). The last alternative is scenario 4, where there are multiple fluctuations in isotopic values between the Griesbachian and Spathian. This incorporates a potential return to oxic seawater values, or at least less anoxic, as well as potential for extremely anoxic conditions, as tracked by Tl isotopic values. As there is extreme variability in the carbon isotopic values of the Early Triassic that likely represents changes in biogeochemical cycles and ocean circulation (Song et al., 2013, 2019), it stands to reason that there may be greater fluctuation in Tl isotopes across this interval. There is also a notable relation between the Tl and carbon isotopic values during the EPME (Newby et al., 2021) that may represent continued covariation throughout the Early Triassic. Scenario 4 may have greater viability due to Tl’s short residence time while still globally homogenous isotopic composition compared to most other global redox proxies employed for the Early Triassic, namely uranium isotopes (Song et al., 2019; F. Zhang et al., 2018; Zhao et al., 2020), allowing for short-term excursions in redox that are only recorded in Tl isotopes. There is also evidence from several local proxies of fluctuations in redox across this interval (Grasby et al., 2013; Song et al., 2012; Wignall et al., 2016; Wu et al., 2024). However, this returns to scenario 2’s biodiversity impact in a far more concerning manner, where rapid changes in redox conditions of the ocean may have a greater likelihood of inducing an extinction that is not properly noted in the remainder of the Early Triassic outside of the SSB (Bond & Grasby, 2017; Foster & Sebe, 2017; Grasby et al., 2016; L. Zhang et al., 2019). Therefore, some other considerations for these scenarios should be taken into account to consider them of greater possibility.

Overall, scenario 1 presents the most straightforward explanation as to how previous and current work on Tl isotopes across the Permo-Triassic transition are connected while scenario 4 presents a more complex but potentially supported alternative. However, even more probable is some degree of overlap between the various scenarios. There are certainly small changes that may occur in Tl, especially around the notable carbon excursions at this time like seen with scenario 4. It is also possible that any of the concerns for minor extinctions due to rapid transitions in Tl isotopes and oceanic redox may be of minimal concern due to the heavy degree of recovery from the EPME limiting the proportion of survival species to go extinct in this interval. Therefore, these scenarios provide guides to connecting the Griesbachian work (Newby et al., 2021) to Smithian data. Taken in conjunction with other proxies across this interval (Grasby et al., 2013; Song et al., 2012, 2019; Wignall et al., 2016; Wu et al., 2024; F. Zhang et al., 2018; Zhao et al., 2020), it seems evident that anoxic conditions are prevalent in the Early Triassic and scenarios that are in greater agreement to that are more likely.

**S2. Supplemental Tables:**

Table S1: Fe speciation duplicate values, bolding duplicated values

| **Sample*** | **Fe (wt%)** |
| --- | --- |
| WAL B 3m (carb) | 0.04 |
| **WAL B 3m (carb)** | **0.04** |
| WAL B 42m (carb) | 0.02 |
| **WAL B 42m (carb)** | **0.02** |
| WAL B 16m (oxide) | 0.31 |
| **WAL B 16m (oxide)** | **0.30** |
| WAL B 60m (oxide) | 0.26 |
| **WAL B 60m (oxide)** | **0.27** |
| WAL B -4m (mag) | 0.25 |
| **WAL B -4m (mag)** | **0.22** |
| WAL B 48m (mag) | 0.22 |
| **WAL B 48m (mag)** | **0.22** |

Table S2: Trace metal geostandard values, bolding reported values

| **Sample*** | **Al (wt%)** | **V (ppm)** | **Mn (ppm)** | **Fe (wt%)** | **Mo (ppm)** | **U (ppm)** |
| --- | --- | --- | --- | --- | --- | --- |
| **SCO-1 (reported)** | **7.235** | **131** | **410.465** | **2.931** | **1.370** | **3** |
| **SCO-1 (measured)** | 6.509 | 115.973 | 413.537 | 2.929 | 1.610 | 4.071 |
| **SDO-1 (reported)** | **6.494** | **160** | **325** | **6.533** | **134** | **48.8** |
| **SDO-1 (measured)** | 6.181 | 161.333 | Undetected† | 6.062 | 141.257 | 49 |
| **SGR-1 (reported)** | **2.678** | **128** | **263.317** | **2.119** | **35.1** | **5.4** |
| **SGR-1 (measured)** | 3.417 | 132.739 | Undetected† | 2.026 | 30.935 | 5.917 |

*reported is reference values, measured is our values

†Machine error caused readings below blank for only Mn on these runs

**S3. Supplemental Figures:**

**Figure S1: Various geochemical components versus thallium isotopes**

Cross-plots of geochemical components of sediments and the potential to influence thallium isotopes. Includes total organic carbon (TOC) vs. ε^205^Tl_auth_ (A), Mn_EF_ vs. ε^205^Tl_auth_ (B), Fe_py_/Fe_HR_ vs. ε^205^Tl_auth_ (C), Mo_EF_ vs. ε^205^Tl_auth_ (D), and Tl_auth_ vs. ε^205^Tl_auth_ (E). TOC values derived from Hammer et al. (2019), with all other data from this study. Lines of regression included as dashed line with R^2^ values listed in each plot.

**S4. References**

Algeo, T. J., & Maynard, J. B. (2004). Trace-element behavior and redox facies in core shales of Upper Pennsylvanian Kansas-type cyclothems. *Chemical Geology*, *206*(3–4), 289–318. https://doi.org/10.1016/j.chemgeo.2003.12.009

Bond, D. P. G., & Grasby, S. E. (2017). On the causes of mass extinctions. *Palaeogeography, Palaeoclimatology, Palaeoecology*, *478*, 3–29. https://doi.org/10.1016/j.palaeo.2016.11.005

Bowman, C. N., Young, S. A., Kaljo, D., Eriksson, M. E., Them, T. R., Hints, O., Martma, T., & Owens, J. D. (2019). Linking the progressive expansion of reducing conditions to a stepwise mass extinction event in the late Silurian oceans. *Geology*, *47*(10), 968–972. https://doi.org/10.1130/G46571.1

Chen, X., Li, S., Newby, S. M., Lyons, T. W., Wu, F., & Owens, J. D. (2021). Iron and manganese shuttle has no effect on sedimentary thallium and vanadium isotope signatures in Black Sea sediments. *Geochimica et Cosmochimica Acta*, *317*, 218–233. https://doi.org/10.1016/j.gca.2021.11.010

Foster, W. J., & Sebe, K. (2017). Recovery and diversification of marine communities following the late Permian mass extinction event in the western Palaeotethys. *Global and Planetary Change*, *155*(July), 165–177. https://doi.org/10.1016/j.gloplacha.2017.07.009

Galfetti, T., Hochuli, P. A., Brayard, A., Bucher, H., Welssert, H., & Vigran, J. O. (2007). Smithian-Spathian boundary event: Evidence for global climatic change in the wake of the end-Permian biotic crisis. *Geology*, *35*(4), 291–294. https://doi.org/10.1130/G23117A.1

Grasby, S. E., Beauchamp, B., Bond, D. P. G., Wignall, P. B., & Sanei, H. (2016). Mercury anomalies associated with three extinction events (Capitanian crisis, Latest Permian extinction and the Smithian/Spathian extinction) in NW Pangea. *Geological Magazine*, *153*(2), 285–297. https://doi.org/10.1017/S0016756815000436

Grasby, S. E., Beauchamp, B., Embry, A., & Sanei, H. (2013). Recurrent Early Triassic ocean anoxia. *Geology*, *41*(2), 175–178. https://doi.org/10.1130/G33599.1

Hammer, Ø., Jones, M. T., Schneebeli-Hermann, E., Hansen, B. B., & Bucher, H. (2019). Are Early Triassic extinction events associated with mercury anomalies? A reassessment of the Smithian/Spathian boundary extinction. *Earth-Science Reviews*, *195*, 179–190. https://doi.org/10.1016/j.earscirev.2019.04.016

Hansen, B. B., Bucher, H. F. R., Schneebeli-Hermann, E., & Hammer, Ø. (2024). Smithian and Spathian palaeontological records of the Vikinghøgda Formation in Central Spitsbergen. *Lethaia*, *57*(1), 1–15. https://doi.org/10.18261/let.57.1.3

Hansen, B. B., Hammer, Ø., & Nakrem, H. A. (2018). Stratigraphy and age of the Grippia niveau bonebed, Lower Triassic Vikinghøgda Formation, Spitsbergen. *Norwegian Journal of Geology*, *98*(2), 175–187. https://doi.org/10.17850/njg98-2-02

Jenkyns, H. C. (2010). Geochemistry of oceanic anoxic events. *Geochemistry, Geophysics, Geosystems*, *11*(3), Q03004. https://doi.org/10.1029/2009GC002788

Kozik, N. P., Young, S. A., Newby, S. M., Liu, M., Chen, D., Hammarlund, E. U., Bond, D. P. G., Them, T. R., & Owens, J. D. (2022). Rapid marine oxygen variability: Driver of the Late Ordovician mass extinction. *Science Advances*, *8*(46), 1–9. https://doi.org/10.1126/sciadv.abn8345

Leu, M., Schneebeli-Hermann, E., Hammer, Ø., Lindemann, F.-J., & Bucher, H. (2024). Spatiotemporal dynamics of nektonic biodiversity and vegetation shifts during the Smithian–Spathian transition: conodont and palynomorph insights from Svalbard. *Lethaia*, *57*(2), 1–19. https://doi.org/10.18261/let.57.2.3

Newby, S. M., Owens, J. D., Schoepfer, S. D., & Algeo, T. J. (2021). Transient ocean oxygenation at end-Permian mass extinction onset shown by thallium isotopes. *Nature Geoscience*, *14*(9), 678–683. https://doi.org/10.1038/s41561-021-00802-4

Nielsen, S. G., Goff, M., Hesselbo, S. P., Jenkyns, H. C., LaRowe, D. E., & Lee, C. T. A. (2011). Thallium isotopes in early diagenetic pyrite - A paleoredox proxy? *Geochimica et Cosmochimica Acta*, *75*(21), 6690–6704. https://doi.org/10.1016/j.gca.2011.07.047

Nielsen, S. G., Rehkämper, M., & Prytulak, J. (2017). Investigation and application of thallium isotope fractionation. *Reviews in Mineralogy and Geochemistry*, *82*(1), 759–798. https://doi.org/10.2138/rmg.2017.82.18

Nielsen, S. G., Wasylenki, L. E., Rehkämper, M., Peacock, C. L., Xue, Z., & Moon, E. M. (2013). Towards an understanding of thallium isotope fractionation during adsorption to manganese oxides. *Geochimica et Cosmochimica Acta*, *117*, 252–265. https://doi.org/10.1016/j.gca.2013.05.004

Ogg, J. G., Chen, Z. Q., Orchard, M. J., & Jiang, H. S. (2020). The Triassic Period. In *Geologic Time Scale 2020*. BV. https://doi.org/10.1016/B978-0-12-824360-2.00025-5

Ostrander, C. M., Heard, A. W., Swanner, E. D., Shu, Y., Zheng, W., Zhao, Y., & Nielsen, S. G. (2025). Thallium isotope cycling in a ferruginous Precambrian ocean analogue. *Geochimica et Cosmochimica Acta*, *390*(December 2024), 264–275. https://doi.org/10.1016/j.gca.2024.12.008

Ostrander, C. M., Owens, J. D., & Nielsen, S. G. (2017). Constraining the rate of oceanic deoxygenation leading up to a Cretaceous Oceanic Anoxic Event (OAE-2: ∼94 Ma). *Science Advances*, *3*(8), e1701020. https://doi.org/10.1126/sciadv.1701020

Owens, J. D. (2019). Application of Thallium Isotopes: Tracking Marine Oxygenation through Manganese Oxide Burial. In *Elements in Geochemical Tracers in Earth System Science* (1st ed., pp. 1–12). Cambridge University Press.

Owens, J. D., Nielsen, S. G., Horner, T. J., Ostrander, C. M., & Peterson, L. C. (2017). Thallium-isotopic compositions of euxinic sediments as a proxy for global manganese-oxide burial. *Geochimica et Cosmochimica Acta*, *213*, 291–307. https://doi.org/10.1016/j.gca.2017.06.041

Poulton, S. W., & Canfeld, D. E. (2011). Ferruginous conditions: A dominant feature of the ocean through Earth’s history. *Elements*, *7*(2), 107–112. https://doi.org/10.2113/gselements.7.2.107

Rehkämper, M., & Nielsen, S. G. (2004). The mass balance of dissolved thallium in the oceans. *Marine Chemistry*, *85*(3–4), 125–139. https://doi.org/10.1016/j.marchem.2003.09.006

Schoepfer, S. D., Henderson, C. M., Garrison, G. H., Foriel, J., Ward, P. D., Selby, D., Hower, J. C., Algeo, T. J., & Shen, Y. (2013). Termination of a continent-margin upwelling system at the Permian-Triassic boundary (Opal Creek, Alberta, Canada). *Global and Planetary Change*, *105*, 21–35. https://doi.org/10.1016/j.gloplacha.2012.07.005

Schoepfer, S. D., Henderson, C. M., Garrison, G. H., & Ward, P. D. (2012). Cessation of a productive coastal upwelling system in the Panthalassic Ocean at the Permian-Triassic Boundary. *Palaeogeography, Palaeoclimatology, Palaeoecology*, *313*–*314*, 181–188. https://doi.org/10.1016/j.palaeo.2011.10.019

Song, H., Du, Y., Algeo, T. J., Tong, J., Owens, J. D., Song, H., Tian, L., Qiu, H., Zhu, Y., & Lyons, T. W. (2019). Cooling-driven oceanic anoxia across the Smithian/Spathian boundary (mid-Early Triassic). *Earth-Science Reviews*, *195*(January), 133–146. https://doi.org/10.1016/j.earscirev.2019.01.009

Song, H., Tong, J., Algeo, T. J., Horacek, M., Qiu, H., Song, H., Tian, L., & Chen, Z. Q. (2013). Large vertical δ13CDIC gradients in Early Triassic seas of the South China craton: Implications for oceanographic changes related to Siberian Traps volcanism. *Global and Planetary Change*, *105*, 7–20. https://doi.org/10.1016/j.gloplacha.2012.10.023

Song, H., Wignall, P. B., Tong, J., Bond, D. P. G., Song, H., Lai, X., Zhang, K., Wang, H., & Chen, Y. (2012). Geochemical evidence from bio-apatite for multiple oceanic anoxic events during Permian-Triassic transition and the link with end-Permian extinction and recovery. *Earth and Planetary Science Letters*, *353*–*354*, 12–21. https://doi.org/10.1016/j.epsl.2012.07.005

Them, T. R., Gill, B. C., Caruthers, A. H., Gerhardt, A. M., Gröcke, D. R., Lyons, T. W., Marroquín, S. M., Nielsen, S. G., João, P. T. A., & Owens, J. D. (2018). Thallium isotopes reveal protracted anoxia during the Toarcian (Early Jurassic) associated with volcanism, carbon burial, and mass extinction. *Proceedings of the National Academy of Sciences of the United States of America*, *115*(26), 6596–6601. https://doi.org/10.1073/pnas.1803478115

Them, T. R., Gill, B. C., Caruthers, A. H., Gröcke, D. R., Tulsky, E. T., Martindale, R. C., Poulton, T. P., & Smith, P. L. (2017). High-resolution carbon isotope records of the Toarcian Oceanic Anoxic Event (Early Jurassic) from North America and implications for the global drivers of the Toarcian carbon cycle. *Earth and Planetary Science Letters*, *459*, 118–126. https://doi.org/10.1016/j.epsl.2016.11.021

Wignall, P. B., Bond, D. P. G., Sun, Y., Grasby, S. E., Beauchamp, B., Joachimski, M. M., & Blomeier, D. P. G. (2016). Ultra-shallow-marine anoxia in an Early Triassic shallow-marine clastic ramp ( Spitsbergen ) and the suppression of benthic radiation. *Geology Magazine*, *153*(2), 316–331. https://doi.org/10.1017/S0016756815000588

Wu, Y., Pohl, A., Tian, L., Corso, J. D., Cui, Y., Chu, D., Tong, J., Song, H., Song, H., & Song, H. (2024). Recurrent marine anoxia in the Paleo-Tethys linked to constriction of seaways during the Early Triassic. *Earth and Planetary Science Letters*, *643*(April), 118882. https://doi.org/10.1016/j.epsl.2024.118882

Zhang, F., Romaniello, S. J., Algeo, T. J., Lau, K. V., Clapham, M. E., Richoz, S., Herrmann, A. D., Smith, H., Horacek, M., & Anbar, A. D. (2018). Multiple episodes of extensive marine anoxia linked to global warming and continental weathering following the latest Permian mass extinction. *Science Advances*, *4*(4), e1602921. https://doi.org/10.1126/sciadv.1602921

Zhang, F., Shen, S. zhong, Cui, Y., Lenton, T. M., Dahl, T. W., Zhang, H., Zheng, Q. feng, Wang, W., Krainer, K., & Anbar, A. D. (2020). Two distinct episodes of marine anoxia during the Permian-Triassic crisis evidenced by uranium isotopes in marine dolostones. *Geochimica et Cosmochimica Acta*, *287*, 165–179. https://doi.org/10.1016/j.gca.2020.01.032

Zhang, L., Orchard, M. J., Brayard, A., Algeo, T. J., Zhao, L., Chen, Z. Q., & Lyu, Z. (2019). The Smithian/Spathian boundary (late Early Triassic): A review of ammonoid, conodont, and carbon-isotopic criteria. *Earth-Science Reviews*, *195*(February), 7–36. https://doi.org/10.1016/j.earscirev.2019.02.014

Zhao, H., Algeo, T. J., Liu, Y., Chen, Z. Q., Zhang, L., Hu, Z., & Li, Z. (2020). Lower Triassic carbonate δ238U record demonstrates expanded oceanic anoxia during Smithian Thermal Maximum and improved ventilation during Smithian-Spathian boundary cooling event. *Palaeogeography, Palaeoclimatology, Palaeoecology*, *539*(388), 109393. https://doi.org/10.1016/j.palaeo.2019.109393
